# Oxidative Stress and Lysosomal Dysfunction in Neurodegenerative Diseases: Underlying Mechanisms and Nanotherapeutic Targeting Strategies

**DOI:** 10.3390/antiox15010073

**Published:** 2026-01-06

**Authors:** Yuhe Hu, Zhaofei Yang, Xu Wang, Xiang Li, Min Wei

**Affiliations:** Key Laboratory of Liaoning Province for Research on the Pathogenic Mechanisms of Neurological Diseases, The First Affiliated Hospital, Dalian Medical University, Dalian 116021, Chinayangzhaofei@dmu.edu.cn (Z.Y.);

**Keywords:** neurodegenerative diseases, oxidative stress, lysosomal dysfunction, nanodrugs

## Abstract

Neurodegenerative diseases (NDDs), defined by the progressive loss of neurons, present a major challenge to global health. Oxidative stress and lysosomal dysfunction are both key pathogenic factors in NDDs, and they do not operate in isolation; instead, the vicious cycle they form, often mediated through organellar crosstalk, serves as the core driver of the pathological progression of NDDs, collectively worsening disease outcomes. Specifically, excessive reactive oxygen species (ROS) can disrupt lysosomal membrane integrity through lipid peroxidation and inhibit the activity of vacuolar ATPase (V-ATPase), ultimately leading to impaired lysosomal acidification. Meanwhile, lysosomal dysfunction hinders the clearance of damaged mitochondria (the primary endogenous source of ROS), toxic protein aggregates, and free iron ions. This further exacerbates ROS accumulation and accelerates neuronal degeneration. Conventional therapeutic approaches have limited efficacy, primarily due to the challenges in crossing the blood–brain barrier (BBB), insufficient targeting ability, and an inability to effectively intervene in this pathological loop. Nanotherapeutics, leveraging their tunable physicochemical properties and modular functional design, represent a transformative strategy to address these limitations. This review systematically elaborates on the reciprocal interplay between oxidative stress and lysosomal dysfunction in NDDs, with a particular focus on the central role of lysosome-mitochondria axis dysfunction, critically appraises recent advances in nanotechnology-based targeted therapies, and thereby provides a comprehensive theoretical framework to guide the development of novel NDD therapeutics.

## 1. Introduction

Neurodegenerative diseases (NDDs) are a group of chronic neurological disorders characterized by the progressive loss of neuronal structure and function, accompanied by abnormal protein deposition, including Alzheimer’s disease (AD), Parkinson’s disease (PD), Huntington’s disease (HD), amyotrophic lateral sclerosis (ALS), and others [1,2]. These diseases not only cause severe deterioration of patients’ cognitive abilities, motor functions or autonomic nervous functions, leading to loss of self-care capacity, but also impose heavy caregiving and economic burdens on families and society. With the acceleration of global population aging, the incidence of NDDs is increasing rapidly, making them one of the most pressing public health challenges worldwide.

The etiology of NDDs is complex and is generally considered to be the result of interactions between genetic susceptibility and environmental factors (such as toxin exposure, trauma, and lifestyle) [3,4]. Different NDDs vary in the brain regions they affect and their primary pathological features, with examples including β-amyloid (Aβ) plaques and neurofibrillary tangles in AD, and α-synuclein-containing Lewy bodies and the loss of dopaminergic neurons in the substantia nigra in PD [5,6]. In-depth studies have identified a series of common, interconnected pathogenic mechanisms that play a core role in disease occurrence and progression [5,7,8]. Among these, oxidative stress and lysosomal dysfunction are regarded as core mechanisms, whose abnormal interaction forms a vicious cycle that acts as a key driver of irreversible pathology in NDDs.

Oxidative stress arises from an imbalance between the production and clearance of reactive oxygen species (ROS) and reactive nitrogen species (RNS) in cells [9,10]. Neurons are particularly sensitive to oxidative damage due to their high oxygen consumption, high content of oxidizable lipids and unsaturated fatty acids, and relatively low antioxidant defense capacity [11]. Excessive ROS/RNS directly attack lipids, proteins, and DNA, disrupt membrane integrity, inactivate key enzymes, cause mitochondrial failure, and ultimately trigger apoptotic or necrotic pathways [12,13]. A large body of evidence indicates that the accumulation of oxidative damage is an early event and a persistent driving factor in NDDs, occurring even years before the appearance of typical clinical symptoms [14,15,16].

Lysosomes serve as the degradation centers and signaling hubs in cells, playing a crucial role in maintaining cellular homeostasis [17]. As a key organelle responsible for degrading macromolecular substances including misfolded proteins and damaged organelles, lysosomes rely on an acidic environment with a pH of 4.5–5.0 and the activity of hydrolases such as cathepsins [18]. In NDDs, lysosomal acidification disorders, decreased lysosomal enzyme activity, or transport defects (such as impaired autophagic flux) all lead to the failure of effective clearance of abnormal protein aggregates and damaged organelles [19,20]. These accumulated toxic substances not only directly induce neurotoxicity but also further exacerbate oxidative stress by promoting the release of more ROS from damaged mitochondria, forming a vicious cycle [21]. Conversely, oxidative stress can also damage lysosomal membrane structure through lipid peroxidation and inhibit hydrolase activity, while lysosomal dysfunction impairs the cell’s ability to clear oxidative damage products, ultimately forming a tight positive feedback loop that collectively accelerates neuronal degeneration [22,23].

Although significant progress has been made in the research on NDDs, the existing treatment methods still have obvious limitations. Current drugs mainly provide palliative relief for symptoms and cannot effectively delay or halt the underlying disease process. Moreover, due to the presence of the blood–brain barrier (BBB), it is difficult to deliver drugs accurately to disease sites, resulting in limited efficacy and increased risk of systemic side effects [24,25]. Therefore, the development of novel therapeutic strategies that can target and regulate oxidative stress and lysosomal dysfunction has become a key direction to overcome current challenges.

Against this background, nanomaterials have shown great potential in the treatment of NDDs due to their unique physicochemical properties, including small-size effect, large specific surface area, and ease of functional modification [26]. Particularly for the aforementioned key pathogenic pathways of oxidative stress and lysosomal dysfunction, nanomaterials offer innovative and attractive solutions. They can act as efficient carriers for antioxidants or exhibit intrinsic antioxidant activity, targeting and scavenging excessive ROS/RNS to protect neurons from oxidative damage [27]. Meanwhile, well-designed nanocarriers can effectively deliver drugs or gene therapy molecules to lysosomes, regulate lysosomal pH, enhance enzyme activity, or promote autophagy, thereby restoring their clearance function [28,29]. In addition, nanomaterials can achieve BBB penetration, targeted accumulation at disease sites, and stimuli-responsive drug release through surface modification, greatly improving the specificity and efficiency of treatment while reducing systemic side effects [30].

This review aims to systematically elaborate on the core pathological mechanisms of NDDs, with a particular focus on the oxidative stress-lysosome axis as a central driver of disease progression. On this basis, it focuses on evaluating the unique advantages, latest research progress, and representative strategies of nanomaterials in intervening in these two key pathogenic pathways, and thereby hopes to provide new ideas and a theoretical basis for the development of more effective NDD treatments.

## 2. Oxidative Stress and NDDs

### 2.1. Sources of ROS and Neurotoxicity

ROS and RNS are generated from both endogenous and exogenous sources, forming a complex reactive species interactome (RSI) where these molecules interact and regulate each other’s production and effects [31]. Within neurons, mitochondria constitute the principal endogenous source. Under normal physiological conditions, ROS such as superoxide anions produced by ETC complexes can be cleared by the mitochondrial antioxidant system such as superoxide dismutase 2 (SOD2) and glutathione peroxidase (GPx) to sustain cellular redox homeostasis [32]. However, in NDDs, electron leakage due to dysfunction of the electron transport chain (ETC) represents a core mechanism of mitochondrial ROS production [33]. Mitochondrial dysfunction causes blocked electron transfer and increased electron leakage. This dysfunction includes mitochondrial cytochrome c oxidase (Complex IV) inhibition by Aβprotein in AD, complex I deficiency in PD, and respiratory chain assembly disruption by mutant huntingtin (mHTT) in HD, and other mechanisms [34,35]. The production of ROS far exceeds the scavenging capacity of the antioxidant system, making mitochondria the main source of ROS in neurons.

Members of the RSI exhibit strong neurotoxicity through interconnected pathways [36]. Excessive ROS and RNS attack biological macromolecules (lipids, proteins, and nucleic acids) in neurons, triggering cascading damage. ROS disrupt unsaturated fatty acids (e.g., arachidonic acid) in membrane phospholipids through hydrogen abstraction reactions, generating toxic aldehyde products such as 4-hydroxynonenal (4-HNE) and malondialdehyde (MDA) [22,37]. This leads to impaired membrane integrity, increased permeability, and ultimately disruption of the intracellular environment. In addition, ROS and RNS modify proteins through carbonylation and nitration, which not only inactivates key enzymes such as lysosomal cathepsins and mitochondrial respiratory chain enzymes but also directly promotes abnormal aggregation and fibrillation of pathogenic proteins such as Aβ, tau, and α-synuclein [15,38]. Moreover, ROS oxidize DNA/RNA to generate products such as 8-hydroxy-2′-deoxyguanosine (8-OHdG), triggering gene mutations or chromosomal damage [39,40]. Combined with mitochondrial failure, this collectively activates the caspase-dependent apoptotic pathway.

### 2.2. Differences in ROS Sources and Pathological Cascade Amplification in Different NDDs

**Figure 1:**
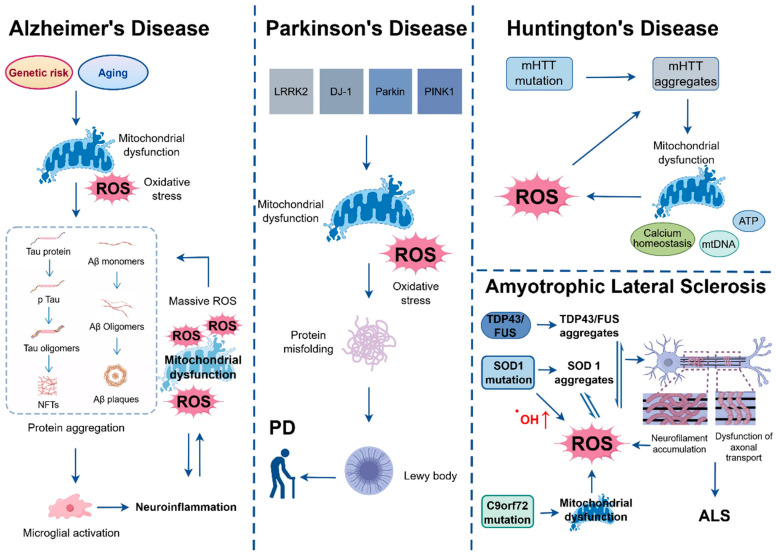
Distinct ROS sources drive disease-specific pathological cascades in major NDDs. (Image created by Figdraw (https://www.figdraw.com)).

In NDDs, although ROS production is a common mechanism, there are significant differences in the pathological events driving ROS generation among different diseases (Figure 1). These differences are closely related to disease-specific pathogenic mutations, core pathological proteins, and affected cell types. Oxidative stress not only directly damages neurons but also drives a series of protein pathological cascade reactions, becoming a core link in disease progression [41].

#### 2.2.1. AD—Protein Aggregation Drives Abundant ROS Production and Pathological Amplification

In AD, the abnormal aggregation of Aβ and hyperphosphorylation of tau are core pathological events that not only drive substantial ROS production but also engage in a vicious cycle with oxidative stress [39]. Genetic risk factors, such as the ApoE4 allele, significantly increase susceptibility to this pathological process by impairing Aβ clearance and exacerbating neuroinflammation [42,43]. Aβ is produced by the cleavage of amyloid precursor protein (APP) by β-secretase (BACE1) and γ-secretase. Oxidative stress promotes the activity of these two secretases, increasing Aβ production [44,45]. At the same time, oxidative stress also induces misfolding and aggregation of Aβ, forming neurotoxic amyloid plaques [46]. These plaques and Aβ oligomers can activate microglia in the brain, triggering a persistent neuroinflammatory response that releases large amounts of inflammatory factors and ROS. The latter further promotes Aβ production and damages neurons. Aβ oligomers can directly bind to neuronal membranes to activate NADPH oxidase and also inhibit Complex IV activity, leading to increased electron leakage; meanwhile, Aβ-induced lysosomal membrane permeabilization (LMP) releases cathepsins, further disrupting mitochondrial function [47,48]. It is important to emphasize that mitochondrial dysfunction is not only a consequence of Aβ toxicity but may also be an initiating event in the early stages of the disease due to aging or genetic factors, and the ROS produced by the mitochondria themselves provide one of the initiating factors for the entire pathological cycle [49]. This ultimately forms a malignant cascade reaction in which protein aggregation, ROS generation, mitochondrial damage, and neuroinflammation promote each other. In addition, the oxidative stress and neuroinflammatory environment synergistically affect the phosphorylation state of tau protein. Under normal physiological conditions, tau maintains microtubule stability through the dynamic balance of phosphorylation and dephosphorylation. However, oxidative stress activates various protein kinases such as glycogen synthase kinase-3β (GSK-3β) and cyclin-dependent kinase 5 (CDK5), leading to tau hyperphosphorylation [50]. Hyperphosphorylated tau dissociates from microtubules, forms neurofibrillary tangles, disrupts the neuronal cytoskeleton, impedes axonal transport and nutrient delivery, and further exacerbates neuronal dysfunction [51].

#### 2.2.2. PD—Gene Mutations and Environmental Toxins Drive ROS Pathology

In PD, the pathological events driving ROS generation are driven by gene mutations and environmental toxins. Mutations in the leucine-rich repeat kinase 2 (*LRRK2*) gene are among the most common genetic causes of both familial and sporadic PD. Among these, the G2019S mutation enhances kinase activity to phosphorylate substrates such as Rab10 and Rab8a. On one hand, this disrupts the balance between the mitochondrial fission protein Drp1 and the fusion protein Mfn2, leading to mitochondrial fragmentation and ROS leakage [52,53]; on the other hand, it interferes with the membrane transport of lysosomal V-ATPase (especially the V0a1 subunit) (see Section 3.2), impairing lysosomal acidification capacity and forming a synergistic pathological effect of mitochondrial damage and impaired lysosomal clearance [54,55]. Mutations in the *PINK1* or *Parkin* (*PRKN*) genes are important causes of early-onset, autosomal recessive familial PD. These mutations disrupt the PINK1-Parkin pathway, leading to continuous accumulation of ROS by blocking mitophagy [56]. Specifically, Parkin mutations result in the loss of its E3 ubiquitin ligase activity, which not only blocks the initiation of mitophagy but also disrupts the dynamic balance of mitochondrial fission-fusion, thereby further expanding the scope of ROS generation [57,58]. Mutations or deletions in the *PARK7/DJ-1* gene are associated with early-onset familial PD. Mutant *DJ-1* loses its antioxidant function and mitochondrial protective effect, which on one hand reduces the cell’s ability to clear ROS, and on the other hand causes mitochondrial membrane damage and abnormal opening of mitochondrial permeability transition pores, accelerating ROS accumulation [59]. In addition, the excessive accumulation of ferrous iron (Fe^2+^) in the substantia nigra catalyzes ROS production through the Fenton reaction, and environmental toxins such as MPTP/rotenone directly inhibit mitochondrial complex I [60,61]. These factors, together with the aforementioned gene mutations, constitute the upstream driving system for ROS generation in PD, and all promote each other with the oxidative aggregation of α-synuclein, further amplifying the effect of ROS accumulation. α-synuclein is a protein expressed at the presynaptic membrane. Under normal conditions, α-synuclein regulates synaptic transmission, whereas under oxidative stress, it undergoes oxidative modification and forms insoluble aggregates known as Lewy bodies [62]. Lewy bodies not only interfere with synaptic transmission and mitochondrial function in dopaminergic neurons but also further inhibit mitophagy, exacerbate ROS generation, and ultimately lead to damage and death of dopaminergic neurons [63].

#### 2.2.3. ALS—Pathogenic Gene Mutations Dominate the ROS Pathological Cascade

In ALS, the pathological events are dominated by pathogenic gene mutations. *C9orf72* is the most common ALS-causing gene. The G_4_C_2_ repeat expansion in the *C9orf72* gene produces dipeptide repeat proteins, which target mitochondria, disrupt membrane potential, and induce ROS release [64]. Meanwhile, loss of *C9orf72* function impairs lysosomal clearance of damaged mitochondria [65]. In addition to *C9orf72*, *SOD1* mutations are also a key factor in ALS pathogenesis, causing approximately 20% of familial ALS cases [66]. Normal SOD1 acts as an antioxidant enzyme, dismutating superoxide anions into hydrogen peroxide (H_2_O_2_). However, most *SOD1* mutations (e.g., A4V, G93A) alter its spatial structure, leading to loss of enzyme activity. This results in the failure to clear superoxide anions, whose accumulation and subsequent conversion into the more toxic H_2_O_2_ and hydroxyl radicals (^•^OH) disrupt cellular redox balance [67]. Additionally, mutant SOD1 may aberrantly bind copper (Cu^2+^) and zinc (Zn^2+^), forming complexes that catalyze the Fenton reaction to convert H_2_O_2_ into ^•^OH, thereby substantially increasing ROS levels [68]. At the same time, mutant SOD1 aggregates into insoluble inclusions, disrupting mitochondrial membrane integrity and inhibiting the activity of respiratory chain complexes, further promoting ROS generation. The above pathological processes collectively drive the pathological phosphorylation of TDP-43 and the formation of inclusions in the cytoplasm [69]. TDP-43 inclusions not only interfere with mitochondrial gene transcription and translation but also inhibit the autophagic pathway, leading to continuous accumulation of ROS and pathological proteins and driving motor neuron degeneration. Emerging evidence also implicates the human endogenous retrovirus K envelope (HERV-K env) protein in ALS pathogenesis, where its expression can induce lysosomal stress and contribute to neuronal toxicity, potentially linked to lysosomal iron dysregulation [70,71,72].

#### 2.2.4. HD—mHTT Drives ROS Generation and Pathological Spread

In HD, the mechanism of ROS generation is mainly driven by mHTT [73]. The core pathological mechanism of HD originates from the expansion of CAG trinucleotide repeats in the *HTT* gene, which leads to abnormal folding and aggregation of mHTT protein with expanded polyglutamine (polyQ) sequences [74]. This impairs mitochondrial function through multiple aspects, including respiratory chain assembly and function, calcium homeostasis regulation, and mitochondrial dynamics, ultimately promoting ROS generation [7,75].

ROS exacerbate the toxicity and pathological spread of mHTT in multiple ways. On one hand, ROS oxidatively modify the cysteine residues of mHTT, enhancing its aggregation ability and promoting the formation of insoluble mHTT aggregates. These aggregates further disrupt mitochondrial function, inhibit ETC complex assembly, interfere with calcium homeostasis, and induce misfolding of normal HTT protein through the “seeding effect”, expanding the pathological scope. On the other hand, ROS inhibit the expression of PGC-1α (a key factor in mitochondrial biogenesis) in the nucleus, downregulate antioxidant genes such as *SOD2* and *SOD1*, and weaken the antioxidant defense capacity of neurons [76,77].

In summary, in different NDDs, the upstream pathological events driving ROS production vary significantly due to differences in the core pathological mechanisms of the diseases, and these differences directly determine the primary and secondary sources of ROS. This specificity is not only an important basis for differences in disease pathological characteristics and clinical phenotypes but also provides directions for precise intervention in diseases. For example, ROS generation in AD can be reduced by targeting Aβ/tau aggregation, ROS in PD can be regulated by repairing mitophagy, and strategies for ALS/HD need to prioritize pathways related to pathogenic gene mutations.

## 3. Lysosomal Dysfunction and Neurodegeneration

The lysosome, a core organelle in eukaryotic cells responsible for degrading biological macromolecules and recycling damaged organelles, must maintain functional homeostasis for cellular metabolic balance, and it is particularly crucial for neurons with high metabolic demands and terminal differentiation [78,79]. Beyond its canonical degradative role, the lysosome is a signaling hub regulating nutrient sensing, metabolism, and cell death pathways such as LMP-mediated apoptosis and ferroptosis [80].

**Figure 2:**
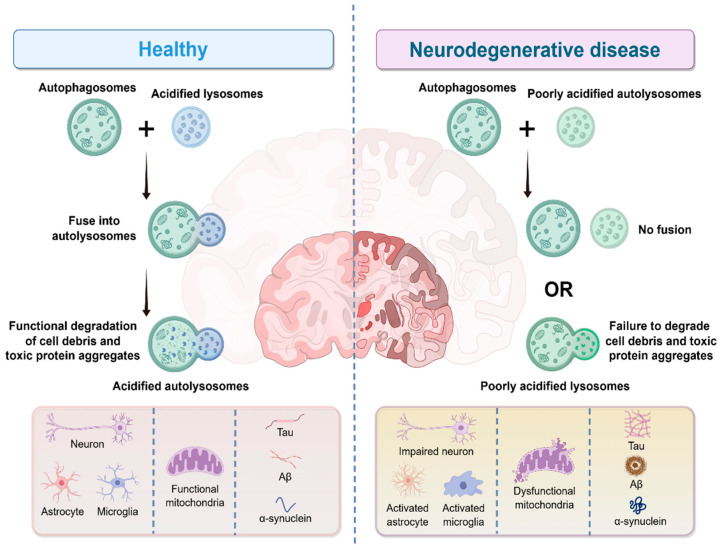
Lysosomal dysfunction in NDDs. (Image created by Figdraw (https://www.figdraw.com/)).

In NDDs, lysosomal dysfunction has been confirmed to be a core link driving pathological progression (Figure 2). From impaired acidification capacity in the early stage to the collapse of degradation function in the late stage, a series of abnormalities lead to the failure of clearance of intracellular metabolic waste (e.g., lipid fragments) and toxic protein aggregates (e.g., Aβ, α-synuclein, tau) [8,81]. This not only directly disrupts the structure and function of neurons but also amplifies pathological effects through glial cell-mediated inflammatory responses, ultimately exacerbating the process of neurodegeneration.

### 3.1. Dysregulation of the Autophagy-Lysosome Pathway

Autophagy encompasses multiple subtypes with distinct mechanisms and substrates, all of which are dysregulated in NDDs [82]. Among these, mitophagy refers to the selective clearance of damaged mitochondria [83]. Lipophagy is responsible for degrading lipid droplets, and its impairment under pathological conditions leads to abnormal lipid accumulation and lipotoxicity [84]. Chaperone-mediated autophagy (CMA), which targets soluble proteins containing the KFERQ motif for lysosomal degradation, is disrupted in AD and PD [85]. Specifically, in AD, tau hyperphosphorylation inhibits CMA by competing with chaperone proteins; in PD, α-synuclein aggregates block CMA receptor (LAMP2A) trafficking to the lysosomal membrane [86,87,88]. Additionally, macroautophagy, which is responsible for the non-selective degradation of cytoplasmic components, is also dysregulated in NDDs, with impaired autophagosome-lysosome fusion further exacerbating the accumulation of toxic protein aggregates [89]. The coordinated dysregulation among these autophagic subtypes collectively aggravates lysosomal dysfunction and oxidative stress, suggesting that future therapeutic interventions should simultaneously target multiple autophagic pathways.

The autophagy-lysosome pathway is the core degradation system for cells to clear abnormal substances and maintain homeostasis. Its complete process includes autophagy initiation, autophagosome formation, autophagosome-lysosome fusion, and substrate degradation [90,91]. Dysregulation at any step can lead to pathological consequences. Autophagy initiation is tightly regulated by the mTOR signaling pathway. Under normal conditions, mTOR inhibits autophagy by phosphorylating downstream targets; when cells face nutrient deficiency or oxidative stress, mTOR activity is inhibited by pathways such as AMPK, and autophagy is subsequently activated [92]. However, in NDDs, pathological factors such as Aβ oligomers and TDP-43 protein continuously activate the PI3K-AKT pathway, leading to abnormal phosphorylation of mTOR. Even when cells are in a stressed state, autophagy cannot be effectively initiated, ultimately resulting in the accumulation of toxic proteins [19,93,94]. On the other hand, the fusion of autophagosomes and lysosomes relies on membrane docking mediated by soluble N-ethylmaleimide-sensitive factor attachment protein receptors (SNARE) proteins such as Syntaxin 17 and VAMP8 to achieve content transport [95]. In AD, tau phosphorylation disrupts the interaction between Syntaxin 17 and lysosomal membrane proteins; in PD, LRRK2 gene mutations interfere with the membrane transport of V-ATPase, impairing lysosomal acidification capacity and indirectly reducing the fusion efficiency of autophagosomes and lysosomes [19,55,93]. It is worth noting that the accumulation of undegraded autophagosomes in neurons occurs in the early stage of the disease, suggesting that it is an early driving event in disease progression [93,96].

### 3.2. Lysosomal Dysfunction in Neurodegeneration

Lysosomal dysfunction in NDDs is not a single defect but involves multi-dimensional disorders of acidification, degradation, membrane integrity, and signaling, showing both common and disease-specific pathological features among different diseases. First, impaired lysosomal acidification is a central early defect. Normal lysosomes rely on V-ATPase to pump protons into the lumen to maintain an acidic environment (pH 4.5–5.0). This process requires the synergistic action of ion channels, including TRPML1, which regulates autophagosome-lysosome fusion via calcium ions (Ca^2+^) release, and TMEM175, which maintains intraluminal electroneutrality via K^+^ efflux [80,97]. In AD, familial mutations in *PSEN1* and *APP* disrupt V-ATPase function, leading to deacidification; *PSEN1*-knockout mice show reduced expression of the V0a1 subunit of V-ATPase, while the APP β-CTF fragment (especially the phosphorylated Tyr^682^ form) can bind to the cytoplasmic V0a1 subunit of V-ATPase, directly inhibiting proton pump activity [98,99]. In PD, mutations in LRRK2 and ATP6AP2 interfere with the membrane transport of V-ATPase, impairing its acidification capacity [54]. In contrast, defects in TMEM175 (a lysosomal K^+^ channel) cause rare “over-acidification”, which, although phenotypically opposite, also inhibits the activity of hydrolases such as cathepsins and promotes α-synuclein aggregation [18,100]. This acidification abnormality occurs in the early stage of the disease. For example, in AD mouse models, the increase in lysosomal pH occurs 5–6 months earlier than the formation of Aβ plaques, and PANTHOS structure can serve as an early pathological marker [96].

In addition, the impaired clearance of lysosomal degradation substrates further amplifies the pathological effect. When lysosomal acidification is abnormal or hydrolase activity decreases, not only are toxic protein aggregates unable to be degraded, but the clearance of damaged organelles like mitochondria is also hindered. In dopaminergic neurons differentiated from iPSCs of PD patients, lysosomal dysfunction leads to blocked mitophagy. ROS released by damaged mitochondria not only disrupts the integrity of the lysosomal membrane through lipid peroxidation, but also induces oxidative stress to exacerbate neuronal apoptosis [101]. At the same time, lysosomal dysfunction in glial cells indirectly damages neurons through inflammatory responses. For example, in AD, microglia cannot degrade phagocytosed Aβ due to lysosomal deacidification; instead, Aβ is released to adjacent neurons through exosomes, accelerating pathological spread [102]. Astrocytes, on the other hand, show reduced expression of glutamate transporters due to lysosomal dysfunction, leading to glutamate toxicity [103].

Furthermore, the disruption of lysosomal membrane integrity is a key link in late-stage pathology and can trigger lysosomal stress responses (LSR), a protective signaling cascade aimed at restoring lysosomal function [104]. However, in the chronic pathological milieu of NDDs, sustained oxidative stress and toxic protein aggregates can overwhelm or dysregulate the LSR, converting it from a protective mechanism into a driver of pathology. To understand this shift, it is essential to dissect the core mechanism of the LSR. Specifically, the core of the LSR involves the activation and nuclear translocation of the transcription factor EB (TFEB), which upregulates the expression of lysosomal biogenesis, autophagy-related genes, and membrane repair proteins to counteract lysosomal damage [104,105]. Recent studies further reveal that proteins such as TMEM55B coordinate the restoration of lysosomal function under oxidative stress by linking autophagic flux, lysosomal repair, and the activation of TFE3 (a member of the TFEB family) [106]. Therefore, the interplay between oxidative stress and lysosomal dysfunction, largely mediated through the LSR, forms a core axis driving the progression of NDDs. Persistent failure of the LSR leads to irreversible loss of lysosomal function and accelerated neuronal death.

Toxic protein aggregates (e.g., Aβ oligomers, α-synuclein aggregates) and ROS released by damaged mitochondria directly disrupt the lipid bilayer structure of lysosomal membranes. In frontotemporal dementia (FTD)/ALS, progranulin (PGRN) deficiency caused by GRN mutations impairs the membrane repair ability of the endosomal sorting complex required for transport (ESCRT) system, ultimately leading to LMP [107]. *C9orf72* risk variants block autophagosome-lysosome fusion through loss of function, and their toxic products (e.g., dipeptide repeat proteins) also directly damage lysosomal membranes [108,109]. *TMEM106B* (transmembrane protein 106B) risk variants abnormally regulate lysosomal membrane transport and morphology, leading to lysosomal expansion and impaired degradation [110]. After membrane leakage, hydrolases such as cathepsins in the lumen are released into the cytoplasm, where they undergo uncontrolled proteolysis of cellular components, including cytoskeletal proteins. At the same time, released toxic fragments (e.g., α-synuclein fragments) induce misfolding of normal proteins through the “seeding effect”, forming a vicious cycle of membrane damage and toxic spread [111,112]. In addition, studies on clinical specimens have also confirmed the pathogenicity of lysosomal dysfunction. In the brain tissue of early AD patients (Braak II stage), lysosomes already show expansion and membrane permeabilization [113]. In the cerebrospinal fluid of early PD patients, the levels of lysosomal marker proteins (LAMP1, cathepsin D) are positively correlated with the content of α-synuclein, suggesting that lysosomal damage can serve as an early biomarker of the disease [114]. Restoring lysosomal acidification (e.g., targeting V-ATPase) or enhancing autophagic flux (e.g., activating TFEB) has become an important strategy for intervening in these diseases.

Notably, beyond its degradative function, the lysosome serves as a key executor of regulated cell death pathways, and its dysfunction is directly linked to neuronal loss. LMP leads to the leakage of proteases such as cathepsins into the cytosol, which can then cleave pro-apoptotic factors like Bid or activate caspases, thereby initiating or amplifying apoptotic pathways [115,116]. Furthermore, the lysosome acts as a central hub for iron storage and the homeostasis of redox-active metal ions [117]. LMP or lysosomal dysfunction results in the release of Fe^2+^ into the cytoplasm, forming a labile iron pool. This pool can catalyze the Fenton reaction, generating highly toxic hydroxyl radicals that drive lipid peroxidation—a hallmark of ferroptosis [118]. Therefore, in NDDs, lysosomal dysfunction not only impairs cellular clearance but also actively drives neurons toward death through multiple interconnected pathways, including LMP-mediated apoptosis and iron-dependent ferroptosis.

### 3.3. Lessons from Lysosomal Storage Diseases: Implications for NDD Therapeutics

Lysosomal storage diseases (LSDs), such as Gaucher disease and Niemann-Pick disease, are caused by mutations in lysosomal enzymes or transporters, leading to substrate accumulation, lysosomal dysfunction, and often neurodegeneration [119]. Interestingly, LSDs share several pathological features with NDDs, including impaired lysosomal acidification, protein aggregation, oxidative stress, and neuroinflammation [120]. This overlap suggests common pathogenic nodes and therapeutic opportunities.

Emerging therapies for LSDs aim to restore lysosomal function. Enzyme replacement therapy (ERT) delivers functional recombinant enzymes (e.g., glucocerebrosidase for Gaucher disease) via intravenous infusion, often requiring strategies to enhance brain delivery [121]. Substrate reduction therapy (SRT) uses small molecules (e.g., eliglustat) to inhibit substrate synthesis [122]. Gene therapy and gene editing approaches seek to correct the underlying genetic defect. It is particularly important to note that when these strategies are combined with nanocarriers capable of crossing the BBB and achieving targeted delivery, they will demonstrate tremendous translational potential. Notably, for Niemann-Pick disease type C (NPC), a disorder caused by mutations in *NPC1* or *NPC2* genes leading to cholesterol accumulation in lysosomes, studies have shown that nanoparticles containing β-cyclodextrin can cross the BBB and mediate cholesterol efflux from the brain, thereby alleviating neuropathological symptoms [123]. These strategies, when combined with nanocarriers for BBB penetration and targeted delivery, hold promise for repurposing in NDDs. For example, nanoparticle-mediated delivery of lysosomal enzymes or gene-editing tools could be adapted to enhance lysosomal function in PD or AD models, where similar enzymes like GBA1 are risk factors [124,125]. Furthermore, molecular chaperone therapy, initially developed for LSDs to stabilize mutant enzymes, could be applied to stabilize lysosomal proteins or even pathological protein conformations in NDDs [126]. Thus, insights and technological platforms from LSD research provide a valuable translational framework for developing lysosome-targeted nanotherapeutics in NDDs.

## 4. Bidirectional Reinforcement Between Oxidative Stress and Lysosomal Damage

Building upon the introductory overview and the details in Section 3, this section will now provide a comprehensive and systematic synthesis of the mechanisms underlying this critical bidirectional interaction, with an emphasis on lysosome-mitochondria crosstalk.

Oxidative stress triggers the collapse of lysosomal function. As the primary target of oxidative damage, lysosomal membranes rich in unsaturated fatty acids undergo lipid peroxidation under ROS attack, generating toxic products such as 4-HNE and MDA, disrupting membrane protein function, and increasing LMP [127,128]. LMP causes proton leakage, disrupting the acidic environment necessary for lysosomal function, and leads to the leakage of hydrolases such as cathepsins into the cytoplasm, which non-specifically degrades intracellular structures and activates apoptotic pathways [112,129,130]. ROS also inhibit the nuclear translocation of the key master regulator TFEB by activating upstream signals such as mTOR [131,132]. This systematically downregulates the transcription of lysosomal biogenesis-related genes (e.g., *LAMP1*, *CTSD*), fundamentally impairing the cell’s repair and clearance capabilities and forming a secondary vicious cycle of functional damage and blocked synthesis.

On the other hand, lysosomal dysfunction also significantly exacerbates oxidative stress. Lysosomes are the endpoint of autophagic flux; their dysfunction (e.g., acidification failure, fusion disorders) directly leads to blocked clearance of damaged mitochondria (due to defective mitophagy) and misfolded protein aggregates. These “cellular wastes” especially dysfunctional mitochondria, continuously produce ROS, further exacerbating oxidative stress [133]. In addition, after LMP, leaked contents such as cathepsin B and iron ions act as endogenous danger signals, efficiently activating the NLRP3 inflammasome [112,129]. The activated inflammasome drives the maturation and secretion of pro-inflammatory factors such as IL-1β and IL-18 in a caspase-1-dependent manner, recruiting and activating microglia [134]. These activated immune cells generate large amounts of ROS through mechanisms such as NADPH oxidase (NOX2), spreading oxidative stress from within affected neurons to the entire neurogenic microenvironment and achieving cascade amplification of damage [135].

Furthermore, lysosomes are the core site of intracellular iron storage, and their stability is crucial for iron homeostasis [136]. The lysosomal iron pool is critical for cellular metabolism but also a source of toxicity upon release [137,138]. Damage to the lysosomal membrane or functional defects can lead to the abnormal leakage of Fe^2+^ [139]. Escaped Fe^2+^ acts as an endogenous danger signal, efficiently activating the NLRP3 inflammasome, which drives neuroinflammation and secondary ROS production by immune cells [140]. More directly, it catalyzes the generation of highly toxic hydroxyl radicals through the Fenton reaction, driving the accumulation of lethal lipid peroxides [141]. When the key repair enzyme GPX4 is continuously inhibited, it will irreversibly trigger iron-dependent programmed cell death, namely ferroptosis [142,143]. This pathway has been confirmed in various NDD models such as AD, PD, and ALS [144,145]. Beyond iron, lysosomes are also a pivotal storage and release site for Ca^2+^ [146,147]. Their dysfunction disrupts Ca^2+^ efflux through channels like TRPML1, impairing mitochondrial calcium signaling, metabolism, and function, thereby creating another route for exacerbating ROS generation [148,149].

Collectively, these interactions coalesce into a central vicious cycle fundamentally driven by lysosome-mitochondria crosstalk. Dysfunctional mitochondria release ROS, which can damage lysosomal membranes via lipid peroxidation, leading to LMP and the leakage of contents including cathepsins and ions. Conversely, impaired lysosomal function disrupts mitophagy, resulting in the accumulation of damaged mitochondria and further ROS production. This reciprocal deterioration constitutes a key driver of disease progression. It is important to emphasize that beyond communication via soluble signaling molecules, lysosomes and mitochondria also engage in physical interaction and direct material exchange through dynamic membrane contact sites. Moreover, beyond communication via soluble signaling molecules, lysosomes and mitochondria also engage in more direct physical interplay. These sites mediate the transfer of metabolites such as Ca^2+^, lipids (e.g., cholesterol), and Fe^2+^, which are crucial for maintaining organelle homeostasis [150,151]. In NDDs, lysosomal dysfunction (e.g., altered pH, increased membrane permeability) can destabilize the number, stability, or function of these contact sites, leading to dysregulated mitochondrial calcium signaling, disrupted iron metabolism, and abnormal lipid deposition, thereby exacerbating mitochondrial dysfunction and oxidative stress. Although the initial pathogenic insults may vary across different NDDs (e.g., exogenous neurotoxins and *PINK1* gene mutations trigger damage at the mitochondrial level), once established, this self-sustaining lysosome-mitochondria vicious cycle amplifies itself and becomes a common, converging pathway driving irreversible progression. Therefore, therapeutic strategies aimed at restoring lysosomal function and its ionic homeostasis represent a crucial approach to intervening in this detrimental cycle.

## 5. Nanotherapeutic Strategies to Disrupt the Oxidative Stress-Lysosome Axis

The intricate crosstalk between oxidative stress and lysosomal dysfunction, as detailed in previous sections, presents both a challenge and an opportunity for therapeutic intervention. The ideal therapeutic agent must not only traverse the BBB but also be specifically activated within this pathological milieu to break the self-reinforcing cycle. Nanotechnology, with its unparalleled tunability in size, surface chemistry, and stimuli-responsiveness, emerges as a powerful platform to meet these demands. This section is structured around the primary therapeutic objectives derived from the pathological mechanisms: (i) scavenging oxidative stress and mitigating its downstream damage, (ii) restoring lysosomal acidification and degradative function, and (iii) implementing multi-targeting strategies for synergistic effects. We will analyze how the design of nanotherapeutics is intelligently tailored to address these distinct yet interconnected goals.

### 5.1. Nanotherapeutic Strategies Targeting Oxidative Stress

This section discusses nanotherapeutic strategies designed to intervene in the oxidative stress pathways detailed in Section 2, which are driven by upstream pathological events such as Aβ/tau in AD, mutant LRRK2 or α-synuclein in PD, and toxic gene products in ALS/HD. The primary goal is to disrupt the downstream cascade of ROS-mediated damage to lipids, proteins, and DNA, thereby protecting neuronal integrity. Nanotherapeutic strategies targeting oxidative stress primarily fall into two categories: First, designing ROS-responsive nanocarriers that utilize the cleavage properties of polymers containing thioketal (TK) or boronate ester bonds that are cleaved in the high-ROS environment of lesions, enabling spatiotemporally controlled delivery of antioxidants (e.g., N-acetylcysteine, idebenone) or neuroprotective peptides; second, developing nanomaterials with intrinsic antioxidant activity or enzyme-mimetic activity. For example, carbon-based nanomaterials (fullerenes, carbon quantum dots) can directly quench free radicals; cerium/manganese oxide nanoparticles (CeO_2_, Mn_3_O_4_) can simulate the activity cycle of SOD/catalase (CAT) to clear ROS; multi-enzyme biomimetic nanozymes (e.g., composite materials with SOD/CAT/GPx activity) can efficiently disrupt ROS signaling. Meanwhile, modification with ligands such as transferrin receptor (TfR) antibodies can further enhance BBB penetration and lesion targeting capabilities.

#### 5.1.1. ROS-Responsive Nanocarrier Modulation Therapeutic Strategies

ROS-responsive nanocarriers are a class of nanosystems that can accurately sense the high-ROS microenvironment (e.g., ^•^OH, H_2_O_2_) at disease sites and achieve drug/gene delivery through structural changes or functional activation [152]. The underlying rationale is to incorporate ROS-sensitive chemical groups (e.g., boronate ester bonds, TK bonds, Se-Se bonds) or dynamically responsive materials into the carrier structure [153,154]. In the normal physiological environment (low ROS), the carrier maintains structural stability to prevent premature drug release. Upon reaching the high-ROS pathological regions of NDDs, the sensitive groups undergo specific cleavage, prompting carrier disintegration or the exposure of targeting/therapeutic moieties, and finally achieving on-demand release. This design addresses a key limitation of traditional drug delivery systems, namely non-specific release, not only enhancing efficacy by improving the enrichment efficiency of therapeutic agents at lesions but also minimizing toxic side effects on normal brain tissue. It is particularly suitable for the pathological feature of localized oxidative stress at NDD lesions, providing an innovative solution to the clinical challenges of low drug delivery efficiency in the brain and high toxic side effects.

In AD therapeutic research, such carriers have demonstrated diverse design ingenuity and application potential. For instance, the 4T1 cell membrane-coated nanomodulator (NM/CM) is designed with cell membrane camouflage and ROS responsiveness as its core features. The outer 4T1 cell membrane enables BBB penetration through natural membrane proteins, while the carrier framework consists of PEG and DSPE linked via a thioketal (TK) bond (DSPE-TK-PEG). In the high-ROS environment of AD-affected brain regions, the TK bond is oxidatively cleaved, releasing curcumin (Cur) and siIFITM3. Cur scavenges excess ROS to alleviate oxidative damage, whereas siIFITM3 enters the cytoplasm and silences the *IFITM3* gene, thereby suppressing abnormal Aβ production [155]. The key advantage of this design is its deep integration of cell membrane camouflage-derived targeting capability and precise drug release from ROS-responsive properties. It not only addresses the core obstacle in AD drug delivery namely BBB penetration but also realizes multi-pathological intervention via dual-drug synergy.

**Figure 3:**
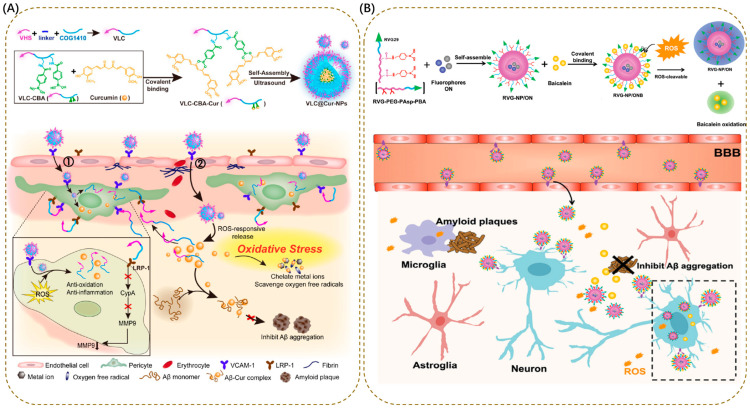
Schematics of ROS-Responsive Nanocarriers for the Treatment of NDDs. (**A**) Schematic diagram of the nanoprodrug VLC@Cur-NPs, depicting its mechanism for rescuing dysfunctional pericytes in AD [156] Copyright © 2024, American Chemical Society; (**B**) Schematic diagram of the theranostic platform RVG-NP/ONB, showing its design and ROS-triggered, dual-channel fluorescence activation and drug release in the AD brain [157] Copyright © 2024 Wiley-VCH GmbH.

In another representative study, the VLC@Cur-NPs nanoprodrug improves treatment specificity through the synergistic design of a fusion peptide (VLC) and ROS-sensitive bonds. VLC consists of VHS (a peptide with high affinity for VCAM-1) and COG1410 (a neuroprotective peptide), and is coupled to Cur via a boronate ester bond. By virtue of the specific binding of the VHS peptide to VCAM-1 (highly expressed on the surface of pericytes), the carrier can accurately target pericyte lesions in AD. In the high-ROS environment, the boronate ester bond is cleaved to release Cur. Cur not only efficiently scavenges ROS and inhibits Aβ aggregation but also synergizes with COG1410 to promote pericyte regeneration, simultaneously improving neurovascular function (Figure 3A) [156]. A major strength of this design is its integration of targeted localization and synergistic therapy: the fusion peptide not only achieves precise anchoring to a key AD pathological site (pericytes) but also covers three core AD pathologies (excessive ROS, Aβ deposition, and neurovascular damage) through the synergistic effect of two active peptides, significantly enhancing the comprehensiveness and synergy of the treatment.

In addition, ROS-responsive nanocarriers have also been extended to the field of “integrated diagnosis and therapy”, providing a new tool for early AD intervention. For example, the RVG-NP/ONB nanotheranostic platform uses PEG-PAsp as a carrier to load an anthracene-based fluorescent probe (N), oligoaniline (O), and the therapeutic drug baicalein (BAI), achieving ROS-responsive release via the cleavage of boronate ester bonds. The carrier surface is modified with the RVG29 peptide to enhance BBB penetration and neuronal targeting capabilities. In early AD lesions with high ROS levels, cleavage of the boronate ester bond promotes the release of BAI, which not only reduces neuronal ROS levels and inhibits Aβ aggregation but also activates dual-channel fluorescence imaging (425 nm blue light and 500 nm green light). In 6-month-old 3xTg-AD mouse models, this platform can accurately identify early lesions in the hippocampus and cortex and reduce Aβ oligomers (Figure 3B) [157]. The core value of this design is embodying a theranostic approach by integrating two key functions. The fluorescent probe offers visual evidence for early AD pathological changes and the therapeutic drug simultaneously delivers intervention. This combination perfectly matches the clinical demand for early AD diagnosis and treatment and provides a strategy to address these challenges.

In summary, through the design of oxidative stress microenvironment sensing-targeted delivery-controlled release, ROS-responsive nanocarriers demonstrate unique advantages in regulating key pathological links of NDDs (such as α-synuclein, Aβ, and tau aggregation, neuroinflammation, mitochondrial dysfunction, and neurovascular damage). They not only overcome the bottleneck of low brain delivery efficiency of traditional drugs but also reduce toxic side effects through on-demand release, providing a highly effective and low-toxicity treatment option for NDDs driven by oxidative stress. Especially in terms of multi-target synergistic intervention and early integrated diagnosis and therapy, such carriers show irreplaceable technical potential. Their design logic is highly compatible with the complex pathological mechanisms of NDDs, and they are expected to break the limitations of the singularity of existing therapeutic approaches, becoming an important direction for future clinical translation of NDD therapies.

#### 5.1.2. Therapeutic Strategies Using Nanomaterials with Intrinsic Antioxidant Activity

The therapeutic strategy using nanomaterials with intrinsic antioxidant activity mainly utilizes the inherent antioxidant properties of nanomaterials or loads antioxidant enzymes to directly scavenge excessive ROS in the body [158,159]. Although natural antioxidant enzymes (e.g., SOD, CAT) can efficiently scavenge ROS, they have limitations such as poor stability, susceptibility to inactivation in physiological conditions, and difficulty in crossing the BBB, restricting their clinical application. In recent years, nanomaterials with intrinsic antioxidant activity have become a key direction to break through the bottlenecks of traditional therapies due to their high stability, tunable biocompatibility, and multi-dimensional intervention capabilities for pathological links. According to differences in antioxidant mechanisms, such materials can be divided into two major categories: “nanozymes” and “non-enzymatic intrinsic antioxidant nanomaterials”. Both can be combined with precise delivery strategies to achieve BBB penetration and lesion targeting, constituting a promising therapeutic strategy for NDDs that simultaneously provides cytoprotection and targets underlying pathological processes. However, there are significant differences in their action pathways and advantageous application scenarios.

##### Nanozymes: Antioxidant Therapy Centered on Enzyme-Mimetic Catalysis

Nanozymes are the most widely studied subclass of nanomaterials with intrinsic antioxidant activity. Their core feature is to achieve efficient and specific scavenging of ROS by simulating the active center structure and catalytic mechanism of natural antioxidant enzymes [160]. By constructing a microenvironment (e.g., metal ion active centers, coordination environments, and spatial conformations) on their surface similar to the active sites of natural enzymes (e.g., SOD, CAT, GPx), these materials reduce the activation energy of ROS conversion, demonstrating advantages such as tunable catalytic activity, high stability, and recyclability [159,161,162]. This characteristic makes them ideal candidates for addressing NDDs.

In terms of action mechanisms, nanozymes can achieve broad-spectrum ROS scavenging through single-enzyme mimicking or multi-enzyme cascade catalysis. For example, 2D vanadium carbide (V_2_C) MXene nanozymes, relying on their layered structure and surface vanadium atom active sites, can simultaneously mimic the activity of enzymes such as SOD, CAT, and peroxidase (POD). Under the action of SOD-like activity, O_2_^•−^ is dismutated into H_2_O_2_ and O_2_; then, through CAT-like activity, H_2_O_2_ is decomposed into harmless H_2_O and O_2_, preventing H_2_O_2_ from being further converted into more toxic ^•^OH; their POD-like activity can also assist in scavenging peroxides in an acidic microenvironment, forming a cascade reaction for ROS scavenging. In MPTP-induced PD mouse models, V_2_C MXene nanozymes can effectively reduce ROS levels in the substantia nigra, maintain the activity of tyrosine hydroxylase (TH), and simultaneously inhibit the expression of the microglial activation marker IBA-1, reducing the release of pro-inflammatory factors IL-1β and TNF-α, and alleviating neuronal apoptosis induced by neuroinflammation [161]. This multi-enzyme cascade design overcomes the limitations of traditional single antioxidants and can systematically regulate the complex ROS network in NDDs. However, the biodegradability and long-term safety of MXene materials remain concerns, as their prolonged retention could pose toxicity risks.

**Figure 4:**
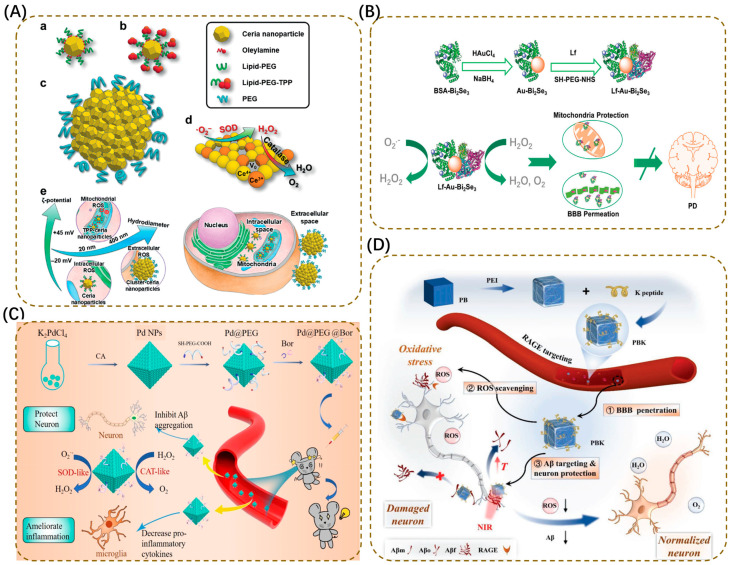
Schematics of nanozyme designs and mechanisms for antioxidant therapy in NDDs. (**A**) Schematic illustration of ceria nanozymes, demonstrating subcellular targeting strategies (a–c), catalytic principles (d), and location-dependent antioxidant functions (e) [163] Copyright © 2018 Wiley-VCH Verlag GmbH & Co. KGaA, Weinheim; (**B**) Schematic illustration of the Lf-Au-Bi_2_Se_3_ nanodots, showing their design and application for targeted PD therapy [164] Copyright © 2021 Wiley-VCH GmbH; (**C**) Schematic diagram of the Pd@PEG@Bor mechanism of AD treatment [165] Copyright © 2021, American Chemical Society; (**D**) Schematic illustration of PBK NPs, highlighting their dual function in disrupting Aβ aggregates and scavenging ROS for AD treatment [166] Copyright © 2023 Wiley-VCH GmbH.

Cerium-based nanozymes are characterized by their precise ROS scavenging ability. Through the redox cycle of Ce^3+^/Ce^4+^, they mimic SOD and CAT activity, efficiently scavenging superoxide radical (O_2_^•−^) and H_2_O_2_. Researchers have designed three differentiated systems to achieve ROS scavenging in different locations: unmodified CeO_2_ nanoparticles (average particle size 3 nm) localize to the cytoplasm after entering cells; Triphenylphosphonium (TPP)-modified CeO_2_ nanoparticles accumulate in mitochondria; and 300 nm cerium-based nanoparticle clusters only scavenge extracellular ROS (Figure 4A). In MPTP mouse models, the groups that scavenged intracellular or mitochondrial ROS significantly inhibited microglial activation, reduced lipid peroxidation, and protected TH activity; in contrast, the group that only scavenged extracellular ROS alleviated neuroinflammation but failed to protect TH activity [163]. This study clarifies the core role of intracellular and mitochondrial ROS in PD pathological progression and also verifies the feasibility of nanozymes for selective ROS scavenging through precise localization.

Novel nanozymes further break through the limitation of single ROS scavenging and expand the regulation of mitochondrial function, blocking ROS regeneration from the source. Platinum-doped cerium dioxide single-atom catalysts (Pt/CeO_2_ SACs), through the synergistic effect of single-atom Pt and Ce^3+^/Ce^4+^, not only scavenge intracellular and extracellular ROS but also consume H^+^ around mitochondria, triggering enhanced mitophagy, and reducing ROS production by clearing damaged mitochondria. After coating with HL-60 cell membranes and modifying with the RVG29 peptide, this system can accurately accumulate around neuronal mitochondria and improve motor disorders in PD mouse models [162]. To further address the issues of brain targeting and enrichment efficiency of nanozymes in PD treatment, lactoferrin (Lf)-modified gold-bismuth selenide nanodots (Lf-Au-Bi_2_Se_3_ NDs) have been designed. They can achieve BBB penetration and neuronal targeting through Lf receptors; after entering cells, they scavenge mitochondrial ROS, reduce α-synuclein phosphorylation, and ultimately increase dopamine content in the striatum of PD mice (Figure 4B) [164].

In AD treatment, the borneol (Bor)-modified octahedral palladium nanozyme (Pd@PEG@Bor) demonstrates promising potential. This system is constructed around an octahedral palladium nanoparticle (Pd NP) core, which exhibits both POD-like and SOD-like enzymatic activities. PEG modification further enhances the material’s biocompatibility and circulatory stability, while borneol effectively improves its BBB penetration capability (Figure 4C) [165]. The nanozyme efficiently scavenges ROS both inside and outside cells, helps maintain mitochondrial membrane potential and calcium ion homeostasis, and reduces the deposition of Aβprotein, thereby offering a novel strategic approach for AD treatment. In addition, chondroitin sulfate-modified MoS_2_ nanomaterials (CS@MoS_2_) utilize the GPx-like activity of MoS_2_ to directly scavenge ROS and upregulate endogenous antioxidant enzymes (SOD/GPx), alleviating Aβ-induced oxidative stress. Furthermore, they inhibit Aβ aggregation and reduce tau hyperphosphorylation by modulating Ca^2+^ homeostasis and suppressing GSK-3β activation, ultimately improving cognitive and anxiety symptoms in AD mice [167]. This synergistic strategy of antioxidant and anti-protein aggregation aligns with the multi-factor pathogenic characteristics of AD and shows significant therapeutic potential. However, the regulation intensity of CS@MoS_2_ on Aβ and tau, as well as its retention time in the brain, may require further optimization.

Prussian blue-based nanozymes enhance AD treatment efficacy through the multi-dimensional integration of antioxidant activity, targeted delivery, and photothermal effects. Prussian blue nanoparticles (PBNPs) exhibit CAT and GPx-like activity due to the Fe^2+^/Fe^3+^ redox pair. After coating with red blood cell (RBC) membranes, they can avoid immune clearance through the CD47 protein, while chelating excessive Cu^2+^ in the brain to inhibit Aβ aggregation. Combined with near-infrared (NIR, 808 nm) light irradiation, their photothermal effect can also disrupt Aβ fibrils [168]. PBK NPs modified with the CKLVFFAED peptide (K peptide) can achieve BBB penetration and Aβ targeting through the RAGE receptor, reducing Aβ deposition and improving cognition in APP/PS1 mouse models (Figure 4D) [166]. This type of design cleverly integrates multiple functional modules, but the tissue penetration depth of NIR light is limited, which may affect the Aβ clearance effect in deep brain tissue. Additionally, the photothermal effect carries the risk of damaging normal cells, requiring strict control of irradiation parameters.

To improve the brain entry efficiency and Aβ targeting of nanozymes in AD treatment, researchers have also developed various novel delivery systems. For example, liposomes co-loading the Aβ-targeting peptide KLVFF and ROS-responsive CeO_2_ nanozymes (KLVFF@LIP-CeO_2_) can enter the brain through the olfactory nerve pathway via intranasal administration, reducing Aβ deposition [169]. Gold nanorods combined with CeO_2_ and modified with the KLVFF peptide can also achieve triple synergy of photothermal effects, antioxidant activity, and anti-Aβ aggregation [170].

Nanozymes provide a revolutionary “drug-free” therapeutic strategy. Compared with natural enzymes, they exhibit superior stability, tunable catalytic activity, and recyclability. Their ability to perform multi-enzyme cascade reactions makes them powerful tools for combating complex ROS networks. However, major limitations include potential loss of enzyme-mimetic activity in biological fluids due to protein corona formation, and long-term biosafety and brain clearance issues of non-biodegradable inorganic nanomaterials. The catalytic specificity and efficiency under physiological conditions also need further improvement. Future research must prioritize the development of biodegradable nanozymes and surface engineering strategies to avoid protein corona formation. In addition, achieving suborganelle-specific targeting (e.g., mitochondria, lysosomes) is crucial for maximizing therapeutic effects and minimizing non-specific catalytic reactions.

##### Non-Enzymatic Intrinsic Antioxidant Nanomaterials: Antioxidant Regulation Centered on Physicochemical Properties

Non-enzymatic intrinsic antioxidant nanomaterials are another important branch of nanomaterials with intrinsic antioxidant activity. Their core feature is that they do not rely on enzyme-mimetic activity but scavenge ROS through inherent properties such as high electron density or specific surface chemistry such as electron transfer ability, free radical scavenging ability, and metal ion chelating ability [171]. These materials do not require simulating the active center structure of natural enzymes, do not follow the kinetics of enzymatic reactions, and are more similar to chemical regulators in their mode of action. They exhibit advantages such as a broad range of action, independence from enzyme inhibitors, and the ability to simultaneously regulate multiple pathological links [172,173]. This characteristic endows these materials with greater application flexibility than nanozymes that rely on specific active centers, especially in pathological microenvironments where enzyme inhibitors are present or ROS types are complex. However, due to the lack of catalytic cycle characteristics, a potential drawback is their typically transient action, which may necessitate higher dosing to maintain efficacy, increasing toxicity risks.

Non-enzymatic intrinsic antioxidant nanomaterials can reduce ROS to harmless species by undergoing electron transfer with ROS via the highly active sites on their surfaces, such as phosphorus and nitrogen doping sites. For example, black phosphorus nanosheets (BPNSs), relying on their layered high specific surface area and high electron cloud density of surface phosphorus atoms, can directly reduce ^•^OH and O_2_^•−^ to H_2_O or O_2_, while being oxidized themselves to biocompatible phosphorus oxides (e.g., H_3_PO_4_). Their reaction rate increases linearly with material concentration, showing no substrate saturation effect, and they can also protect mitochondria. In hA53T α-synuclein transgenic PD mice, BPNSs can activate the autophagic pathway, promote the degradation of abnormal α-synuclein, increase the number of TH-positive neurons in the substantia nigra, and improve the movement distance and exploratory behavior of mice in the open field test [172]. Some non-enzymatic materials can also combine photothermal effects to enhance pathological intervention. For example, macrophage membrane-modified MoS_2_ quantum dots (MoS_2_ QDs/MM) have high electron cloud density at Mo and S atoms on their surface, which can directly undergo electron transfer with ^•^OH and O_2_^•−^ to reduce them to harmless species. At the same time, MoS_2_ QDs can generate photothermal effects under NIR irradiation, which not only disrupt the β-sheet structure of Aβ fibrils but also promote their own ROS scavenging efficiency [174].

Such nanomaterials can also clear ROS by combining free radical scavenging and metal ion chelation. For example, N-doped carbon dots (CDs) form an electronic conjugated structure through doped sites such as pyridinic nitrogen and pyrrolic nitrogen; this structure can directly capture ROS, disperse the energy of free radicals to the entire molecular skeleton via the conjugated system, and reduce their reactivity. Additionally, the pyridinic nitrogen sites on their surface can specifically chelate excessive Fe^2+^ in the brain, preventing Fe^2+^ from reacting with H_2_O_2_ through the Fenton reaction to generate ^•^OH. Following modification with PEG-Lf, a nanosystem (hereafter abbreviated as CPL) is obtained. This nanosystem not only reversibly opens the BBB by releasing NO but also further enhances brain enrichment efficiency by combining with Lf-mediated receptor transport. In PD mouse models, CPL can reduce the production of the lipid peroxidation product MDA, downregulate the expression of the pro-inflammatory cytokine TNF-α, and synergistically improve motor symptoms and neuroinflammation [173].

Iron chelation represents a distinctive yet related non-enzymatic strategy that interferes with the oxidative stress-lysosome axis by targeting the lysosomal iron pool. As a potent iron chelator, deferoxamine (DFO) has demonstrated neuroprotective effects in models of NDDs. Its mechanisms include alleviating lysosomal iron overload, inhibiting Fenton reaction-driven ROS generation, and attenuating ferroptosis [175,176]. Studies have confirmed that intranasal delivery of DFO can reduce iron deposition in models of AD and PD [177,178]. To further enhance the brain delivery and achieve sustained release of DFO, nanocarriers such as polydopamine-modified black phosphorus nanosheets and polymer-based nanoparticles have been explored for DFO delivery, showing improved brain targeting and controlled-release properties [179,180].

In general, non-enzymatic antioxidants avoid the catalytic complexity of nanozymes and act as powerful chemical regulators through direct electron transfer, free radical scavenging, or metal ion chelation. This mechanism endows them with broad-spectrum ROS scavenging ability and independence from specific enzymatic pathways. A significant drawback is their typically stoichiometric (non-catalytic) nature, meaning they are consumed during the process. This may necessitate higher doses to maintain efficacy, thereby increasing potential toxicity risks. Under special conditions such as high oxygen tension, some materials may also exhibit pro-oxidative effects, exacerbating oxidative damage. In addition, the long-term in vivo fate of some carbon-based materials remains to be fully elucidated, raising concerns about potential brain accumulation, requiring longer-term animal experiments to evaluate their biosafety.

### 5.2. Lysosomal Function-Repairing Nanodrugs

Aiming to rectify the core lysosomal impairments outlined in Section 3, including insufficient acidification due to V-ATPase dysfunction, loss of membrane integrity (LMP), and reduced hydrolase activity, this section explores therapeutic strategies based on lysosomal function-repairing nanodrugs. As mentioned earlier, lysosomal dysfunction is one of the core pathological features of NDDs. It is mainly manifested as insufficient lysosomal acidification, abnormal membrane permeability, and reduced hydrolase activity, which directly lead to the failure of effective degradation of neurotoxic proteins such as Aβ, tau, and α-synuclein, thereby triggering protein aggregation and neuronal damage. In this context, it is crucial to understand the cellular uptake and lysosomal fate of nanoparticles. Most nanoparticles enter cells via endocytosis and ultimately localize to lysosomes. Consequently, their intrinsic physicochemical properties directly determine whether they act as a “stress inducer” that exacerbates damage or a “functional module” that assists in homeostasis within the lysosomal environment. Against this background, lysosomal function-repairing nanodrugs, with their core advantages of targeted delivery, controlled release, and biocompatibility, have become a key strategy for restoring lysosomal function and promoting the clearance of toxic proteins. Compared with traditional small-molecule drugs, their targeting and controlled release properties can reduce off-target effects and interference with normal cells, making them more suitable for the precise treatment of NDDs in the brain. Such nanodrugs mainly restore the normal physiological functions of lysosomes by regulating lysosomal pH, enhancing the activity of lysosomal enzyme activity, or promoting lysosomal regeneration [181].

The endocytic uptake of these nanoparticles is a critical first step, determining their intracellular trafficking and fate [182]. Surface properties (such as charge and functional groups), size and shape dictate whether nanoparticles are directed to lysosomes or other compartments [183,184]. It is noteworthy that many inorganic nanomaterials represented by silica nanoparticles, which also enter lysosomes via endocytosis, act as persistent “foreign bodies” due to their non-degradability. Their long-term physical retention abnormally consumes the energy of lysosomal proton pumps, leading to elevated pH, enzyme inactivation, and even membrane damage with content leakage, thereby exacerbating disease pathology [185,186,187]. In contrast, biodegradable nanoparticles, especially those with acidic properties, demonstrate significant advantages. They can gradually degrade within lysosomes, avoiding accumulation. Moreover, their acidic products or buffering capacity do not disrupt acidification; instead, they help stabilize the pH environment, supporting hydrolytic enzyme activity and the completion of autophagic flux [188,189]. Such nanomaterials can themselves act as “active therapeutics” to exert neuroprotective effects while providing an ideal platform for delivering other therapeutic molecules.

**Figure 5:**
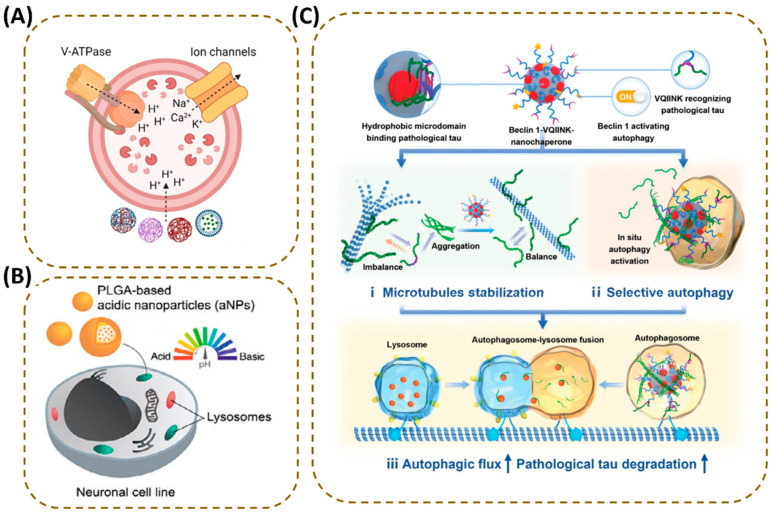
Schematics of nanodrugs designed to restore lysosomal function in NDDs. (**A**) Schematic representation of lysosomal acidification modulation by acidic nanoparticles [93]; (**B**) Schematic diagram of PLGA-based acidic nanoparticles (PLGA-aNPs), illustrating their delivery into neuronal cells [189]; (**C**) Schematic diagram of the Beclin1-VQIINK nanochaperone (nChap), showing its design and mechanism for targeted tau clearance via lysosomal pathway regulation [192] Copyright © 2024 Wiley-VCH GmbH.

Acidic nanoparticles (aNPs) can directly deliver “acid” to lysosomes (Figure 5A) [93]. For example, aNPs using poly(lactic-co-glycolic acid) (PLGA) as a carrier can localize to lysosomes through neuronal endocytosis, and the acidic products released during degradation can directly reverse the lysosomal alkalization state (Figure 5B) [189]. In PD models, PLGA-aNPs can reduce the pH of damaged lysosomes to the physiological range, not only restoring the activity of hydrolases such as cathepsins B and L but also reducing LMP and decreasing the leakage of cathepsin D into the cytoplasm, ultimately achieving inhibition of α-synuclein aggregation and protection of dopaminergic neurons in the substantia nigra [189,190]. In addition, PLGA nanoemulsions can also enhance membrane stability and promote the fusion efficiency of autophagosomes and lysosomes by regulating the cholesterol level of lysosomal membranes. They significantly reduce Aβ plaque deposition in AD models, further verifying the multi-effectiveness of PLGA-based nanodrugs in repairing lysosomal function [191].

In addition to PLGA-based nanodrugs, other types of lysosomal repair nanodrugs also show targeted efficacy, among which nanoparticles with tetrafluorosuccinic acid (TFSA) as the core active component are an important direction. For example, TFSA-based acidic nanoparticles have stronger acidity (pKa ≈ 1.6), resulting in more efficient lysosomal acidification repair: after entering lysosomes, they can be rapidly degraded and release TFSA, which not only directly reduces intraluminal pH but also upregulates the expression of lysosomal V-ATPase subunits, enhancing the active acidification capacity of lysosomes [188]. Similarly, acid-activated acidic nanoparticles (acNPs) based on fluorinated polyesters improve targeting safety through pH-responsive design; these nanoparticles remain stable in the neutral plasma environment (pH 7.4) and only start to degrade and release TFSA when entering dysfunctional lysosomes with pH ≈ 6, restoring lysosomal function through a dual mechanism: on the one hand, directly releasing acidic substances to reduce lysosomal pH, and on the other hand, upregulating V-ATPase expression to enhance active acidification capacity [181]. Although these two studies have not directly verified their efficacy in NDDs models, the pathological features they target such as lipotoxicity-induced insufficient lysosomal acidification and autophagic flux disorder are highly consistent with the core mechanisms of lysosomal dysfunction in NDDs. Moreover, designs like pH responsiveness can be directly applied to brain-targeted delivery scenarios, laying a solid theoretical and technical foundation for their subsequent application in NDDs.

Targeting the specificity of pathological mechanisms in ALS, nanoparticles encapsulating phosphatidylinositol derivatives exert their effects by long-term activation of the lysosomal TRPML1 channel (activation of the TRPML1 channel can promote Ca^2+^ release from lysosomes and regulate lysosomal transport and hydrolase release). In ALS mouse models, this nanodrug can reduce lysosomal damage in motor neurons, decrease the aggregation of mutant SOD1 protein, and significantly improve the motor function and survival period of mice [193].

Nanodrugs can also indirectly repair lysosomes by precisely regulating lysosome-related autophagic pathways, achieving selective clearance of specific toxic proteins. In AD, long-term tau accumulation and autophagic disorders damage lysosomal membrane integrity, leading to reduced expression of lysosome-associated membrane proteins (e.g., LAMP1) and weakened fusion ability. The nanochaperone-based strategy (Beclin1-VQIINK-nChap) involves modifying nanoparticle surfaces with the KFERQ motif, a substrate recognition sequence for CMA, which can target and bind pathogenic tau protein and guide it to enter lysosomes for degradation through the CMA pathway. The Beclin1 peptide on its surface can accurately activate autophagy in tau aggregation regions, promoting the formation of autophagosomes encapsulating pathological tau. This not only provides lysosomes with specific degradation substrates but also avoids the problem of excessive consumption of lysosomal hydrolases and disruption of lysosomal homeostasis caused by traditional non-selective autophagy activators. After treatment with Beclin1-VQIINK-nChap, the expression of LAMP1 in nerve cells returns to normal, lysosomal membrane integrity is significantly improved, and the interaction of fusion-related proteins (e.g., Rab7, VAMP8) is enhanced. In tauopathy mouse models, this nanochaperone can significantly reduce the level of p-tau in the brain, decrease the formation of neurofibrillary tangles, and improve the cognitive function of mice, avoiding the risk of normal protein degradation that may be caused by traditional non-selective autophagy activation (Figure 5C) [192].

In addition, nanodrugs can also be used as carrier platforms to expand lysosomal repair strategies. First, delivering lysosomal function modulators, such as TFEB transcription factor agonists, V-ATPase modulators (e.g., C381, EN6), or autophagy inducers (e.g., rapamycin), through liposomes or polymer nanoparticles to improve their BBB penetration and intracellular delivery efficiency [93,194,195]. Second, developing lysosomal repair-type nanoparticles, such as targeted carriers carrying cholesterol chelators or enzyme replacement therapy molecules (for *GBA1* mutation-related diseases), or designing proton donor-containing polymers to buffer lysosomal pH and restore the acidic environment. In addition, to optimize lysosomal targeting efficiency, lysosome-targeting peptides (e.g., Cysteine-Glutamine peptide) can be modified on the surface or the surface charge of nanoparticles can be regulated (positive charge enhances lysosomal uptake) to ensure that drugs are accurately delivered to the lysosomal compartment.

Taken together, these studies demonstrate that lysosomal function-restoring nanodrugs with different designs can exert their effects by targeting the pathological features of different NDDs through diverse mechanisms, such as acidification restoration, membrane stability regulation, channel activation, and selective autophagy modulation. They provide diversified solutions for the disease-modifying therapy of such diseases, while also laying a foundation for the precise design and clinical translation of subsequent nanodrugs. The core concept lies in the ingenious design of nanomaterials that internalize therapeutic functions, such as providing an acidic microenvironment or activating specific channels, within the carrier itself. This approach unifies the roles of “carrier” and “drug,” enabling more direct and efficient intervention at the subcellular organelle level. Future designs could further focus on the precise modulation of the LSR, for example, by utilizing nanocarriers to deliver TFEB agonists or molecules that regulate its activity, thereby reconstructing lysosomal homeostasis at the transcriptional level and providing new strategies for intervention. However, most current drugs are still in the preclinical stage, and breakthroughs are still needed in terms of long-term brain safety and multi-mechanism synergistic repair capabilities.

### 5.3. Smart Nanodrugs with Multi-Targeting Strategies

Smart nanodrugs with a multi-targeting strategy refer to a class of smart nanodrugs that simultaneously target multiple pathological mechanisms such as oxidative stress, lysosomal dysfunction and inflammation, or simultaneously target multiple disease sites. These nanodrugs typically possess multifunctional surface modifications and responsive release properties, enabling precise drug release and synergistic therapy based on changes in the microenvironment of disease sites. They effectively address the limitation that traditional single-target drugs struggle to cope with the complex pathological networks of NDDs.

Taking PD as an example, its core pathology involves abnormal α-synuclein aggregation and neuroinflammation. Based on this, researchers have developed a nanosystem in which glucose- and trehalose-functionalized carbonized polymer dots (GT-PCDs) load plasmid DNA (pDNA). Relying on its ROS-responsive properties, this system promotes efficient nuclear entry of pDNA in the oxidative stress environment of PD lesions, reducing de novo α-synuclein production by silencing the *SNCA* gene; meanwhile, it facilitates the nuclear translocation of TFEB to restore autophagic flux, accelerating the degradation of preformed α-synuclein aggregates. In addition, glucose modified on the surface enhances BBB permeability through transcytosis mediated by glucose transporter 1, improving the efficiency of drug enrichment in the brain. Ultimately, it significantly alleviates motor deficits and reduces neuroinflammation in MPTP-induced PD mouse models [196]. The core advantage of this system is that it utilizes ROS responsiveness for drug release, reduces α-synuclein production via *SNCA* silencing, and simultaneously restores autophagy to accelerate α-synuclein clearance—this not only enhances therapeutic efficacy at disease sites but also reduces the required drug dosage and associated toxicity.

**Figure 6:**
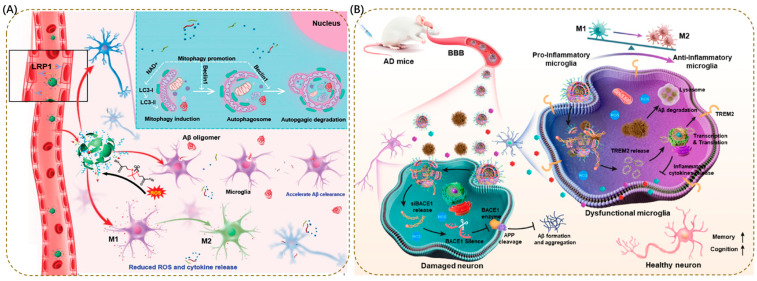
Schematics of multitargeted nanodrugs for synergistic therapy of NDDs. (**A**) Schematic illustration of the P@NB nanoscavenger for inducing autophagy/mitophagy to ameliorate cognitive deficits in AD [198] Copyright © 2023 Wiley-VCH GmbH; (**B**) Schematic illustration of TSEL hybrid nanovesicles for ROS-triggered co-delivery of siBACE1 and pTREM2 to achieve synergistic AD treatment [199] Copyright © 2024, American Chemical Society.

In the field of AD treatment, ROS-responsive nanocarriers enable multi-targeted therapy. For example, the TT-NM/Rapa micellar system, constructed from a TK-bridged PEG-PCL copolymer and functionalized with the TPL targeting peptide, delivers rapamycin to neuronal lesions. Under high-ROS conditions, rapamycin (Rapa) is released to activate autophagy, promoting the clearance of Aβ and p-tau proteins, which alleviates neuronal damage and memory deficits in AD mice [197]. Another type of P@NB nanoscavenger can release NAD+ and Beclin1 in a high-ROS environment: on one hand, it reduces the source of ROS production by activating mitophagy; on the other hand, it accelerates Aβ degradation by inducing the polarization of microglia to the anti-inflammatory M2 phenotype (Figure 6A) [198].

ROS-sensitive drug release strategies are also often combined with inflammation regulation mechanisms, with a typical example being the biomimetic exosome-liposome hybrid nanovesicles (TSEL). These vesicles utilize ROS-sensitive liposomes containing Se-Se bonds to co-deliver siBACE1 and TREM2 plasmid (pTREM2), while integrating the homing ability of exosomes and the BBB-penetrating function of the angiopep-2 peptide. In the high-ROS microenvironment of AD lesions, the vesicles disintegrate and release the drugs: pTREM2 reprograms microglia from the pro-inflammatory M1 to the anti-inflammatory M2 phenotype, enhancing Aβ clearance; simultaneously, siBACE1 knocks down BACE1 to reduce Aβ production. Ultimately, this synergistically improves cognitive impairment in APP/PS1 mouse models (Figure 6B) [199].

In summary, multitargeted nanotherapeutic strategies effectively overcome the limitations of traditional single-target drugs by synergistically intervening in the complex pathological networks of NDDs. Specifically, temporally sequential responsive systems can cascade response to pathological signals, such as first responding to ROS to release antioxidants and then responding to the acidic environment of lysosomes to release functional modulators; Multifunctional integrated nanoparticles can simultaneously carry ROS scavengers and lysosomal modulators, while integrating BBB-penetrating ligands and neuron-targeting molecules; Theranostic integrated designs incorporate MRI/fluorescence imaging capabilities to enable real-time monitoring of the therapeutic process; Biomimetic nanocarriers (e.g., cell membrane-coated carriers) can significantly prolong in vivo circulation time and enhance drug accumulation efficiency at disease sites—all these designs provide novel ideas for the efficient treatment of NDDs.

## 6. Conclusions and Future Perspectives

NDDs, represented by AD, PD, ALS and HD, pose an increasingly severe global public health burden amid population aging. This review systematically elucidates the core pathological mechanisms underlying NDD progression, with a particular focus on the bidirectional vicious cycle and organellar crosstalk (especially lysosome-mitochondria) between oxidative stress and lysosomal dysfunction—we conceptualize this bidirectional vicious cycle as the core pathological axis of NDD progression (rather than isolated pathways), an integrated perspective that has rarely been systematically synthesized in previous reviews, which serves as a central driver of irreversible neuronal degeneration. Excessive ROS not only directly damages neuronal lipids, proteins, and nucleic acids but also disrupts lysosomal membrane integrity and impairs acidification; conversely, lysosomal dysfunction hinders the clearance of damaged organelles and toxic protein aggregates, further amplifying ROS generation and disrupting iron homeostasis. This intertwined regulatory network, involving the RSI and LSR, exacerbates pathological cascades such as protein aggregation, neuroinflammation, and ferroptosis, forming an intractable barrier to traditional therapeutic interventions.

Traditional drugs for NDDs remain limited to palliative symptom management, failing to target the root pathological processes, while the BBB further restricts drug delivery efficiency and amplifies systemic side effects. In this context, nanomaterials have emerged as a transformative platform to address these bottlenecks, leveraging their tunable physicochemical properties and versatile functionalization capabilities. As summarized in this review and Table 1, nanotherapeutic strategies have achieved remarkable progress in disrupting the oxidative stress-lysosome axis: For oxidative stress targeting, ROS-responsive nanocarriers enable spatiotemporally controlled release of antioxidants, while intrinsic antioxidant nanomaterials (e.g., CeO_2_ nanozymes, V_2_C MXene, black phosphorus nanosheets) efficiently scavenge ROS through enzyme-mimetic catalysis or direct electron transfer and iron chelation, with subcellular targeting further enhancing therapeutic specificity and reducing off-target effects; For lysosomal function repair, acid-responsive nanocarriers (e.g., PLGA-based acidic nanoparticles, TFSA-loaded systems) reverse lysosomal alkalization, restore hydrolase activity, and stabilize lysosomal membranes; strategies such as TRPML1 channel activation and CMA regulation also selectively clear toxic aggregates while preserving lysosomal homeostasis; Multitargeted smart nanosystems integrate BBB penetration, stimuli-responsive release, and synergistic intervention, effectively addressing the complex pathological networks of NDDs. Insights from lysosomal storage diseases further enrich the therapeutic toolkit available for adaptation to NDDs.

Despite these advances, the clinical translation of nanotherapeutics faces several critical challenges. These include biosafety concerns, such as the long-term retention and poor biodegradability of inorganic nanomaterials in the brain, and potential off-target toxicities, and the nuanced role of endocytosis where some particles may cause harm while others confer protection. Furthermore, targeting precision needs improvement, as current approaches for BBB penetration and subcellular localization still exhibit insufficient efficiency and consistency. Finally, the pathological complexity of NDDs demands more comprehensive interventions, since single-pathway targeting cannot fully reverse the multifaceted degeneration in advanced stages. The actual efficacy of many nanotherapeutics beyond preclinical models and their potential adverse effects require more thorough discussion and rigorous evaluation.

Looking forward, future research should prioritize: First, developing biodegradable and biocompatible nanomaterials to ensure long-term safety; second, enhancing spatiotemporal precision through advanced targeting strategies and real-time monitoring via theranostic integration; and third, designing adaptive nanosystems that respond to dynamic pathological signals (e.g., sequential responsiveness to ROS and lysosomal pH) for smarter, context-dependent drug release. In parallel, future research should integrate insights from LSD therapies and iron biology to develop novel lysosome-centric interventions. Finally, future research should conduct rigorous long-term toxicity and efficacy studies in relevant animal models and advancing towards standardized manufacturing for clinical trials. By addressing these challenges, nanotechnology holds immense promise to break the oxidative stress-lysosome vicious cycle, paving the way for disease-modifying therapies that fundamentally alter the course of NDDs.

## Figures and Tables

**Table 1:** Summary of Nanotherapeutic Strategies Targeting the Oxidative Stress-Lysosome Axis in NDDs.

Strategy Category	Carrier/Material Type	Targeted Pathology/Target	Cargo/Active Component	Disease Model	Main Effects	Limitations	References
**ROS-Responsive Nanocarriers**							
	Cell membrane-coated nanoregulator (NM/CM)	Brain high ROS microenvironment, *IFITM3* gene	Curcumin (Cur), siIFITM3	AD model	Crosses BBB, scavenges ROS, inhibits Aβ generation	Complex design, long-term safety needs evaluation	[155]
	Fusion peptide prodrug nanoparticle (VLC@Cur-NPs)	Pericytes (VCAM-1), high ROS microenvironment	Fusion peptide (VHS + COG1410), Curcumin (Cur)	AD model	Targets pericytes, scavenges ROS, inhibits Aβ aggregation, promotes neurovascular repair	High target specificity may limit broad application	[156]
	Theranostic nanoplatform (RVG-NP/ONB)	Neurons (RVG29 peptide), high ROS microenvironment	Baicalein (BAI), fluorescent probes	3xTg-AD model	Early lesion fluorescence imaging, simultaneous ROS scavenging, reduces Aβ oligomers	Integration of diagnostic components may affect drug loading capacity	[157]
**Intrinsic Antioxidant Nanomaterials**							
*Nanozymes*	V2C MXene nanosheets	Multiple ROS (O_2_^−^, H_2_O_2_, ^•^OH)	Intrinsic (Multi-enzyme mimicking: SOD, CAT, POD)	MPTP-induced PD model	Cascade ROS scavenging, alleviates neuroinflammation, protects neurons	Biodegradability unclear, potential toxicity from long-term retention	[161]
	Ceria nanoparticles (CeO_2_ NPs)	Intracellular/Mitochondrial/Extracellular ROS	Intrinsic (SOD/CAT mimicking activity)	MPTP-induced PD model	Selective ROS scavenging in specific compartments, protects dopaminergic neurons	Catalytic efficiency and specificity need optimization	[163]
	Platinum-doped ceria single-atom catalyst (Pt/CeO_2_ SACs)	Mitochondria, ROS	Intrinsic (ROS scavenging, induces mitophagy)	PD model	Scavenges ROS, clears damaged mitochondria, improves motor function	Complex synthesis, high cost	[162]
	Lactoferrin-modified Gold-Bismuth Selenide nanodots (Lf-Au-Bi_2_Se_3_ NDs)	Neurons (Lf receptor), mitochondrial ROS	Intrinsic (Antioxidant)	PD model	Targets neurons, scavenges mitochondrial ROS, reduces α-syn phosphorylation, increases dopamine	Complex composition, in vivo metabolic pathways need clarification	[164]
	Chondroitin sulfate-modified Molybdenum Disulfide (CS@MoS_2_)	ROS, Aβ, Tau protein	Intrinsic (Peroxidase-like activity)	AD model	Scavenges ROS, inhibits Aβ aggregation, reduces Tau phosphorylation, improves cognition	Efficacy against Aβ/Tau and brain retention time need optimization	[167]
	Prussian Blue nanoparticles (PBNPs, PBK NPs)	Aβ (KLVFF peptide), ROS, Cu^2+^	Intrinsic (CAT/GPx mimicking, Cu^2+^ chelation, photothermal effect)	APP/PS1 AD model	Scavenges ROS, chelates Cu^2+^ inhibiting Aβ aggregation, NIR photothermal disruption of Aβ fibrils	Limited NIR tissue penetration depth, photothermal parameters require precise control to avoid damage	[166,168]
*Non-Enzymatic*	Black Phosphorus Nanosheets (BPNSs)	ROS, α-syn aggregates	Intrinsic (Direct electron transfer for radical scavenging)	hA53T α-syn transgenic PD model	Scavenges ROS, degrades α-syn, activates autophagy, protects neurons, improves motor function	Prone to oxidation in vivo, stability is a challenge	[172]
	N-doped Carbon Dots (CPL)	ROS, Fe^2+^	Intrinsic (Radical trapping, Fe^2+^ chelation), NO release	PD model	Scavenges ROS, inhibits Fenton reaction, transiently opens BBB for enhanced brain delivery, reduces inflammation	Stoichiometric consumption, may require higher doses, potential toxicity risk	[173]
	Macrophage membrane-modified MoS_2_ Quantum Dots (MoS_2_ QDs/MM)	ROS, Aβ	Intrinsic (Electron transfer for ROS scavenging, photothermal effect)	AD model	Scavenges ROS, photothermal disruption of Aβ fibrils	Long-term biosafety requires further study	[174]
**Lysosomal Function Restoration**							
	Poly(lactic-co-glycolic acid) acidic nanoparticles (PLGA-aNPs)	Lysosomes (via endocytosis)	Acidic degradation products	MPTP-induced PD model	Restores lysosomal acidic pH, enhances hydrolase activity, stabilizes lysosomal membrane, reduces α-syn aggregation	Primarily preclinical, brain entry efficiency needs optimization	[189,190]
	Phosphatidylinositol derivative-loaded nanoparticles	Lysosomal TRPML1 ion channel	Phosphatidylinositol derivative	ALS mouse model	Activates TRPML1, improves lysosomal function, reduces mutant SOD1 aggregation, extends survival	Disease-specific, general applicability needs examination	[193]
	Nano-chaperone (Beclin1-VQIINK-nChap)	Pathological Tau protein, Chaperone-Mediated Autophagy (CMA) pathway	KFERQ motif, Beclin1 peptide	Tauopathy mouse model	Selectively directs pathological Tau to lysosomes via CMA for degradation, restores lysosomal membrane integrity	Targets specific pathological protein, complex design	[192]
**Multi-Targeting Strategies**							
	Glucose/Trehalose-functionalized Carbon Dots (GT-PCDs)	Neurons, high-ROS environment, nucleus	Plasmid DNA (pDNA, targeting *SNCA* gene)	MPTP-induced PD model	ROS-responsive pDNA release, silences *SNCA* to reduce α-syn production, activates TFEB to restore autophagy	Gene delivery efficiency and long-term expression stability need attention	[196]
	ROS-responsive targeted micelles (TT-NM/Rapa)	AD lesion neurons (TPL peptide), high ROS	Rapamycin (Rapa)	3xTg-AD model	Targets neurons, ROS-responsive Rapa release activates autophagy, clears Aβ and p-Tau	Reversal effect on advanced pathology might be limited	[197]
	Nanoscavenger (P@NB)	High ROS microenvironment	NAD^+^, Beclin1	AD model	Releases NAD^+^ to activate mitophagy, releases Beclin1 to induce microglial M2 polarization for Aβ clearance	Precise control of multi-component synergy is challenging	[198]
	Biomimetic nanovesicles (TSEL)	High ROS microenvironment, microglia	siBACE1, pTREM2	APP/PS1 AD model	ROS-responsive cargo release, siBACE1 reduces Aβ production, pTREM2 reprograms microglia to anti-inflammatory M2 phenotype	Risk of immunogenicity and off-target effects for gene vectors	[199]

## Data Availability

No new data were created or analyzed in this study. Data sharing is not applicable to this article.

## References

[1] Erkkinen M.G., Kim M.O., Geschwind M.D. (2018). Clinical Neurology and Epidemiology of the Major Neurodegenerative Diseases. Cold Spring Harb. Perspect. Biol..

[2] Bourdenx M., Koulakiotis N.S., Sanoudou D., Bezard E., Dehay B., Tsarbopoulos A. (2017). Protein aggregation and neurodegeneration in prototypical neurodegenerative diseases: Examples of amyloidopathies, tauopathies and synucleinopathies. Prog. Neurobiol..

[3] Li K., Shacham E., Brown D., Blake M., Zhu Y., Trani J.F., Babulal G.M. (2025). Association of environmental exposome and cognitive function among older adults with and without preclinical Alzheimer’s disease. Alzheimer’s Dement..

[4] Gubert C., Kong G., Renoir T., Hannan A.J. (2020). Exercise, diet and stress as modulators of gut microbiota: Implications for neurodegenerative diseases. Neurobiol. Dis..

[5] Dugger B.N., Dickson D.W. (2017). Pathology of Neurodegenerative Diseases. Cold Spring Harb. Perspect. Biol..

[6] Esmaeili Y., Yarjanli Z., Pakniya F., Bidram E., Łos M.J., Eshraghi M., Klionsky D.J., Ghavami S., Zarrabi A. (2022). Targeting autophagy, oxidative stress, and ER stress for neurodegenerative disease treatment. J. Control. Release.

[7] Klemmensen M.M., Borrowman S.H., Pearce C., Pyles B., Chandra B. (2024). Mitochondrial dysfunction in neurodegenerative disorders. Neurotherapeutics.

[8] Nixon R.A., Rubinsztein D.C. (2024). Mechanisms of autophagy-lysosome dysfunction in neurodegenerative diseases. Nat. Rev. Mol. Cell Biol..

[9] Jomova K., Raptova R., Alomar S.Y., Alwasel S.H., Nepovimova E., Kuca K., Valko M. (2023). Reactive oxygen species, toxicity, oxidative stress, and antioxidants: Chronic diseases and aging. Arch. Toxicol..

[10] Sies H., Mailloux R.J., Jakob U. (2024). Author Correction: Fundamentals of redox regulation in biology. Nat. Rev. Mol. Cell Biol..

[11] Ding X.S., Gao L., Han Z., Eleuteri S., Shi W., Shen Y., Song Z.Y., Su M., Yang Q., Qu Y. (2023). Ferroptosis in Parkinson’s disease: Molecular mechanisms and therapeutic potential. Ageing Res. Rev..

[12] Yang W.S., Stockwell B.R. (2016). Ferroptosis: Death by Lipid Peroxidation. Trends Cell Biol..

[13] Park S.Y., Kim K.Y., Gwak D.S., Shin S.Y., Jun D.Y., Kim Y.H. (2024). L-Cysteine mitigates ROS-induced apoptosis and neurocognitive deficits by protecting against endoplasmic reticulum stress and mitochondrial dysfunction in mouse neuronal cells. Biomed. Pharmacother..

[14] Nunomura A., Perry G. (2020). RNA and Oxidative Stress in Alzheimer’s Disease: Focus on microRNAs. Oxid. Med. Cell Longev..

[15] Perluigi M., Di Domenico F., Butterfield D.A. (2024). Oxidative damage in neurodegeneration: Roles in the pathogenesis and progression of Alzheimer disease. Physiol. Rev..

[16] Lovell M.A., Soman S., Bradley M.A. (2011). Oxidatively modified nucleic acids in preclinical Alzheimer’s disease (PCAD) brain. Mech. Ageing Dev..

[17] Yang C., Wang X. (2021). Lysosome biogenesis: Regulation and functions. J. Cell Biol..

[18] Zhou N., Chen J., Hu M., Wen N., Cai W., Li P., Zhao L., Meng Y., Zhao D., Yang X. (2025). SLC7A11 is an unconventional H^+^ transporter in lysosomes. Cell.

[19] Udayar V., Chen Y., Sidransky E., Jagasia R. (2022). Lysosomal dysfunction in neurodegeneration: Emerging concepts and methods. Trends Neurosci..

[20] Wei M., Li W., Bao G., Yang Z., Li S., Le W. (2025). PM2.5 exposure exacerbates Alzheimer’s disease pathology through lysosomal dysfunction in APP/PS1 mice. Ecotoxicol. Environ. Saf..

[21] Picca A., Calvani R., Coelho-Junior H.J., Landi F., Bernabei R., Marzetti E. (2020). Mitochondrial Dysfunction, Oxidative Stress, and Neuroinflammation: Intertwined Roads to Neurodegeneration. Antioxidants.

[22] Qi Z., Yang W., Xue B., Chen T., Lu X., Zhang R., Li Z., Zhao X., Zhang Y., Han F. (2024). ROS-mediated lysosomal membrane permeabilization and autophagy inhibition regulate bleomycin-induced cellular senescence. Autophagy.

[23] Martelli F., Hernandes N.H., Zuo Z., Wang J., Wong C.O., Karagas N.E., Roessner U., Rupasinghe T., Robin C., Venkatachalam K. (2022). Low doses of the organic insecticide spinosad trigger lysosomal defects, elevated ROS, lipid dysregulation, and neurodegeneration in flies. eLife.

[24] Wu D., Chen Q., Chen X., Han F., Chen Z., Wang Y. (2023). The blood-brain barrier: Structure, regulation, and drug delivery. Signal Transduct. Target. Ther..

[25] Pedder J.H., Sonabend A.M., Cearns M.D., Michael B.D., Zakaria R., Heimberger A.B., Jenkinson M.D., Dickens D. (2025). Crossing the blood-brain barrier: Emerging therapeutic strategies for neurological disease. Lancet Neurol..

[26] Furtado D., Björnmalm M., Ayton S., Bush A.I., Kempe K., Caruso F. (2018). Overcoming the Blood-Brain Barrier: The Role of Nanomaterials in Treating Neurological Diseases. Adv. Mater..

[27] Zhang Y., Zhang Y., Liang R., Zou J., Pei R., Chen X. (2025). Targeted ROS Scavenging for Disease Therapies Using Nanomaterials. Adv. Mater..

[28] Zeng J., Shirihai O.S., Grinstaff M.W. (2019). Degradable Nanoparticles Restore Lysosomal pH and Autophagic Flux in Lipotoxic Pancreatic Beta Cells. Adv. Healthc. Mater..

[29] Placci M., Giannotti M.I., Muro S. (2023). Polymer-based drug delivery systems under investigation for enzyme replacement and other therapies of lysosomal storage disorders. Adv. Drug Deliv. Rev..

[30] Zha S., Liu H., Li H., Li H., Wong K.L., All A.H. (2024). Functionalized Nanomaterials Capable of Crossing the Blood-Brain Barrier. ACS Nano.

[31] Malard E., Valable S., Bernaudin M., Pérès E., Chatre L. (2021). The Reactive Species Interactome in the Brain. Antioxid. Redox Signal.

[32] Zhuang A., Yang C., Liu Y., Tan Y., Bond S.T., Walker S., Sikora T., Laskowski A., Sharma A., de Haan J.B. (2021). SOD2 in skeletal muscle: New insights from an inducible deletion model. Redox Biol..

[33] Reiter R.J., Sharma R.N., Manucha W., Rosales-Corral S., Almieda Chuffa L.G., Loh D., Luchetti F., Balduini W., Govitrapong P. (2024). Dysfunctional mitochondria in age-related neurodegeneration: Utility of melatonin as an antioxidant treatment. Ageing Res. Rev..

[34] Wang W., Ma X., Bhatta S., Shao C., Zhao F., Fujioka H., Torres S., Wu F., Zhu X. (2023). Intraneuronal β-amyloid impaired mitochondrial proteostasis through the impact on LONP1. Proc. Natl. Acad. Sci. USA.

[35] Flønes I.H., Toker L., Sandnes D.A., Castelli M., Mostafavi S., Lura N., Shadad O., Fernandez-Vizarra E., Painous C., Pérez-Soriano A. (2024). Mitochondrial complex I deficiency stratifies idiopathic Parkinson’s disease. Nat. Commun..

[36] Cortese-Krott M.M., Koning A., Kuhnle G.G.C., Nagy P., Bianco C.L., Pasch A., Wink D.A., Fukuto J.M., Jackson A.A., van Goor H. (2017). The Reactive Species Interactome: Evolutionary Emergence, Biological Significance, and Opportunities for Redox Metabolomics and Personalized Medicine. Antioxid. Redox Signal.

[37] Wei M., Bao G., Li S., Yang Z., Cheng C., Le W. (2022). PM2.5 exposure triggers cell death through lysosomal membrane permeabilization and leads to ferroptosis insensitivity via the autophagy dysfunction/p62-KEAP1-NRF2 activation in neuronal cells. Ecotoxicol. Environ. Saf..

[38] Stadtman E.R. (2001). Protein oxidation in aging and age-related diseases. Ann. N. Y. Acad. Sci..

[39] Yu N., Pasha M., Chua J.J.E. (2024). Redox changes and cellular senescence in Alzheimer’s disease. Redox Biol..

[40] Di Minno A., Turnu L., Porro B., Squellerio I., Cavalca V., Tremoli E., Di Minno M.N. (2016). 8-Hydroxy-2-Deoxyguanosine Levels and Cardiovascular Disease: A Systematic Review and Meta-Analysis of the Literature. Antioxid. Redox Signal.

[41] Bai R., Guo J., Ye X.Y., Xie Y., Xie T. (2022). Oxidative stress: The core pathogenesis and mechanism of Alzheimer’s disease. Ageing Res. Rev..

[42] Yin Z., Rosenzweig N., Kleemann K.L., Zhang X., Brandão W., Margeta M.A., Schroeder C., Sivanathan K.N., Silveira S., Gauthier C. (2023). APOE4 impairs the microglial response in Alzheimer’s disease by inducing TGFβ-mediated checkpoints. Nat. Immunol..

[43] Haney M.S., Pálovics R., Munson C.N., Long C., Johansson P.K., Yip O., Dong W., Rawat E., West E., Schlachetzki J.C.M. (2024). APOE4/4 is linked to damaging lipid droplets in Alzheimer’s disease microglia. Nature.

[44] Lee H.J., Jung Y.H., Choi G.E., Kim J.S., Chae C.W., Lim J.R., Kim S.Y., Yoon J.H., Cho J.H., Lee S.J. (2021). Urolithin A suppresses high glucose-induced neuronal amyloidogenesis by modulating TGM2-dependent ER-mitochondria contacts and calcium homeostasis. Cell Death Differ..

[45] Coyle J.T., Puttfarcken P. (1993). Oxidative stress, glutamate, and neurodegenerative disorders. Science.

[46] Calvo-Rodriguez M., Kharitonova E.K., Snyder A.C., Hou S.S., Sanchez-Mico M.V., Das S., Fan Z., Shirani H., Nilsson K.P.R., Serrano-Pozo A. (2024). Real-time imaging of mitochondrial redox reveals increased mitochondrial oxidative stress associated with amyloid β aggregates in vivo in a mouse model of Alzheimer’s disease. Mol. Neurodegener..

[47] Abramov A.Y., Canevari L., Duchen M.R. (2004). Beta-amyloid peptides induce mitochondrial dysfunction and oxidative stress in astrocytes and death of neurons through activation of NADPH oxidase. J. Neurosci..

[48] Sarkar C., Jones J.W., Hegdekar N., Thayer J.A., Kumar A., Faden A.I., Kane M.A., Lipinski M.M. (2020). PLA2G4A/cPLA2-mediated lysosomal membrane damage leads to inhibition of autophagy and neurodegeneration after brain trauma. Autophagy.

[49] Li Y., Xia X., Wang Y., Zheng J.C. (2022). Mitochondrial dysfunction in microglia: A novel perspective for pathogenesis of Alzheimer’s disease. J. Neuroinflamm..

[50] Ai J., Wang H., Chu P., Shopit A., Niu M., Ahmad N., Tesfaldet T., Wang F.H., Fang J.N., Li X. (2021). The neuroprotective effects of phosphocreatine on Amyloid Beta 25-35-induced differentiated neuronal cell death through inhibition of AKT /GSK-3β /Tau/APP /CDK5 pathways in vivo and vitro. Free Radic. Biol. Med..

[51] Drummond E., Pires G., MacMurray C., Askenazi M., Nayak S., Bourdon M., Safar J., Ueberheide B., Wisniewski T. (2020). Phosphorylated tau interactome in the human Alzheimer’s disease brain. Brain.

[52] Keeney M.T., Rocha E.M., Hoffman E.K., Farmer K., Di Maio R., Weir J., Wagner W.G., Hu X., Clark C.L., Castro S.L. (2024). LRRK2 regulates production of reactive oxygen species in cell and animal models of Parkinson’s disease. Sci. Transl. Med..

[53] Wauters F., Cornelissen T., Imberechts D., Martin S., Koentjoro B., Sue C., Vangheluwe P., Vandenberghe W. (2020). LRRK2 mutations impair depolarization-induced mitophagy through inhibition of mitochondrial accumulation of RAB10. Autophagy.

[54] Wallings R., Connor-Robson N., Wade-Martins R. (2019). LRRK2 interacts with the vacuolar-type H^+^-ATPase pump a1 subunit to regulate lysosomal function. Hum. Mol. Genet..

[55] Henry A.G., Aghamohammadzadeh S., Samaroo H., Chen Y., Mou K., Needle E., Hirst W.D. (2015). Pathogenic LRRK2 mutations, through increased kinase activity, produce enlarged lysosomes with reduced degradative capacity and increase ATP13A2 expression. Hum. Mol. Genet..

[56] Kritskaya K.A., Fedotova E.I., Berezhnov A.V. (2024). Impaired Mitochondrial Network Morphology and Reactive Oxygen Species Production in Fibroblasts from Parkinson’s Disease Patients. Biomedicines.

[57] Zilocchi M., Colugnat I., Lualdi M., Meduri M., Marini F., Corasolla Carregari V., Moutaoufik M.T., Phanse S., Pieroni L., Babu M. (2020). Exploring the Impact of PARK2 Mutations on the Total and Mitochondrial Proteome of Human Skin Fibroblasts. Front. Cell Dev. Biol..

[58] Antico O., Thompson P.W., Hertz N.T., Muqit M.M.K., Parton L.E. (2025). Targeting mitophagy in neurodegenerative diseases. Nat. Rev. Drug Discov..

[59] Liu L.L., Han Y., Zhang Z.J., Wang Y.Q., Hu Y.W., Kaznacheyeva E., Ding J.Q., Guo D.K., Wang G.H., Li B. (2023). Loss of DJ-1 function contributes to Parkinson’s disease pathogenesis in mice via RACK1-mediated PKC activation and MAO-B upregulation. Acta Pharmacol. Sin..

[60] Wang Q., Liu J., Zhang Y., Li Z., Zhao Z., Jiang W., Zhao J., Hou L., Wang Q. (2024). Microglial CR3 promotes neuron ferroptosis via NOX2-mediated iron deposition in rotenone-induced experimental models of Parkinson’s disease. Redox Biol..

[61] Lee J., Hyun D.H. (2023). The Interplay between Intracellular Iron Homeostasis and Neuroinflammation in Neurodegenerative Diseases. Antioxidants.

[62] Koeglsperger T., Rumpf S.L., Schließer P., Struebing F.L., Brendel M., Levin J., Trenkwalder C., Höglinger G.U., Herms J. (2023). Neuropathology of incidental Lewy body & prodromal Parkinson’s disease. Mol. Neurodegener..

[63] Mahul-Mellier A.L., Burtscher J., Maharjan N., Weerens L., Croisier M., Kuttler F., Leleu M., Knott G.W., Lashuel H.A. (2020). The process of Lewy body formation, rather than simply α-synuclein fibrillization, is one of the major drivers of neurodegeneration. Proc. Natl. Acad. Sci. USA.

[64] Onesto E., Colombrita C., Gumina V., Borghi M.O., Dusi S., Doretti A., Fagiolari G., Invernizzi F., Moggio M., Tiranti V. (2016). Gene-specific mitochondria dysfunctions in human TARDBP and C9ORF72 fibroblasts. Acta Neuropathol. Commun..

[65] Beckers J., Tharkeshwar A.K., Van Damme P. (2021). C9orf72 ALS-FTD: Recent evidence for dysregulation of the autophagy-lysosome pathway at multiple levels. Autophagy.

[66] McCampbell A., Cole T., Wegener A.J., Tomassy G.S., Setnicka A., Farley B.J., Schoch K.M., Hoye M.L., Shabsovich M., Sun L. (2018). Antisense oligonucleotides extend survival and reverse decrement in muscle response in ALS models. J. Clin. Investig..

[67] Brasil A.A., Magalhães R.S.S., De Carvalho M.D.C., Paiva I., Gerhardt E., Pereira M.D., Outeiro T.F., Eleutherio E.C.A. (2018). Implications of fALS Mutations on Sod1 Function and Oligomerization in Cell Models. Mol. Neurobiol..

[68] Bakavayev S., Chetrit N., Zvagelsky T., Mansour R., Vyazmensky M., Barak Z., Israelson A., Engel S. (2019). Cu/Zn-superoxide dismutase and wild-type like fALS SOD1 mutants produce cytotoxic quantities of H_2_O_2_ via cysteine-dependent redox short-circuit. Sci. Rep..

[69] Jeon G.S., Shim Y.M., Lee D.Y., Kim J.S., Kang M., Ahn S.H., Shin J.Y., Geum D., Hong Y.H., Sung J.J. (2019). Pathological Modification of TDP-43 in Amyotrophic Lateral Sclerosis with SOD1 Mutations. Mol. Neurobiol..

[70] Li W., Lee M.H., Henderson L., Tyagi R., Bachani M., Steiner J., Campanac E., Hoffman D.A., von Geldern G., Johnson K. (2015). Human endogenous retrovirus-K contributes to motor neuron disease. Sci. Transl. Med..

[71] Halcrow P.W., Quansah D.N.K., Kumar N., Steiner J.P., Nath A., Geiger J.D. (2024). HERV-K (HML-2) Envelope Protein Induces Mitochondrial Depolarization and Neurotoxicity via Endolysosome Iron Dyshomeostasis. J. Neurosci..

[72] Küry P., Nath A., Créange A., Dolei A., Marche P., Gold J., Giovannoni G., Hartung H.P., Perron H. (2018). Human Endogenous Retroviruses in Neurological Diseases. Trends Mol. Med..

[73] Song S., Su Z., Kon N., Chu B., Li H., Jiang X., Luo J., Stockwell B.R., Gu W. (2023). ALOX5-mediated ferroptosis acts as a distinct cell death pathway upon oxidative stress in Huntington’s disease. Genes Dev..

[74] Wang N., Zhang S., Langfelder P., Ramanathan L., Gao F., Plascencia M., Vaca R., Gu X., Deng L., Dionisio L.E. (2025). Distinct mismatch-repair complex genes set neuronal CAG-repeat expansion rate to drive selective pathogenesis in HD mice. Cell.

[75] Yablonska S., Ganesan V., Ferrando L.M., Kim J., Pyzel A., Baranova O.V., Khattar N.K., Larkin T.M., Baranov S.V., Chen N. (2019). Mutant huntingtin disrupts mitochondrial proteostasis by interacting with TIM23. Proc. Natl. Acad. Sci. USA.

[76] Paul B.D., Snyder S.H. (2019). Impaired Redox Signaling in Huntington’s Disease: Therapeutic Implications. Front. Mol. Neurosci..

[77] Joshi D.C., Chavan M.B., Gurow K., Gupta M., Dhaliwal J.S., Ming L.C. (2025). The role of mitochondrial dysfunction in Huntington’s disease: Implications for therapeutic targeting. Biomed. Pharmacother..

[78] Zhang Z., Yue P., Lu T., Wang Y., Wei Y., Wei X. (2021). Role of lysosomes in physiological activities, diseases, and therapy. J. Hematol. Oncol..

[79] Ballabio A., Bonifacino J.S. (2020). Lysosomes as dynamic regulators of cell and organismal homeostasis. Nat. Rev. Mol. Cell Biol..

[80] Lawrence R.E., Zoncu R. (2019). The lysosome as a cellular centre for signalling, metabolism and quality control. Nat. Cell Biol..

[81] Nixon R.A. (2024). Autophagy-lysosomal-associated neuronal death in neurodegenerative disease. Acta Neuropathol..

[82] Fleming A., Bourdenx M., Fujimaki M., Karabiyik C., Krause G.J., Lopez A., Martín-Segura A., Puri C., Scrivo A., Skidmore J. (2022). The different autophagy degradation pathways and neurodegeneration. Neuron.

[83] Katayama H., Hama H., Nagasawa K., Kurokawa H., Sugiyama M., Ando R., Funata M., Yoshida N., Homma M., Nishimura T. (2020). Visualizing and Modulating Mitophagy for Therapeutic Studies of Neurodegeneration. Cell.

[84] Haidar M., Loix M., Vanherle S., Dierckx T., Vangansewinkel T., Gervois P., Wolfs E., Lambrichts I., Bogie J.F.J., Hendriks J.J.A. (2022). Targeting lipophagy in macrophages improves repair in multiple sclerosis. Autophagy.

[85] Choi Y.J., Nam Y.A., Hyun J.Y., Yu J., Mun Y., Yun S.H., Lee W., Park C.J., Han B.W., Lee B.H. (2025). Impaired chaperone-mediated autophagy leads to abnormal SORT1 (sortilin 1) turnover and CES1-dependent triglyceride hydrolysis. Autophagy.

[86] Caballero B., Bourdenx M., Luengo E., Diaz A., Sohn P.D., Chen X., Wang C., Juste Y.R., Wegmann S., Patel B. (2021). Acetylated tau inhibits chaperone-mediated autophagy and promotes tau pathology propagation in mice. Nat. Commun..

[87] Kuo S.H., Tasset I., Cheng M.M., Diaz A., Pan M.K., Lieberman O.J., Hutten S.J., Alcalay R.N., Kim S., Ximénez-Embún P. (2022). Mutant glucocerebrosidase impairs α-synuclein degradation by blockade of chaperone-mediated autophagy. Sci. Adv..

[88] Tang F.L., Erion J.R., Tian Y., Liu W., Yin D.M., Ye J., Tang B., Mei L., Xiong W.C. (2015). VPS35 in Dopamine Neurons Is Required for Endosome-to-Golgi Retrieval of Lamp2a, a Receptor of Chaperone-Mediated Autophagy That Is Critical for α-Synuclein Degradation and Prevention of Pathogenesis of Parkinson’s Disease. J. Neurosci..

[89] Rusmini P., Cortese K., Crippa V., Cristofani R., Cicardi M.E., Ferrari V., Vezzoli G., Tedesco B., Meroni M., Messi E. (2019). Trehalose induces autophagy via lysosomal-mediated TFEB activation in models of motoneuron degeneration. Autophagy.

[90] Kuchitsu Y., Taguchi T. (2024). Lysosomal microautophagy: An emerging dimension in mammalian autophagy. Trends Cell Biol..

[91] Klionsky D.J., Abdel-Aziz A.K., Abdelfatah S., Abdellatif M., Abdoli A., Abel S., Abeliovich H., Abildgaard M.H., Abudu Y.P., Acevedo-Arozena A. (2021). Guidelines for the use and interpretation of assays for monitoring autophagy (4th edition). Autophagy.

[92] Guo H., Ouyang Y., Yin H., Cui H., Deng H., Liu H., Jian Z., Fang J., Zuo Z., Wang X. (2022). Induction of autophagy via the ROS-dependent AMPK-mTOR pathway protects copper-induced spermatogenesis disorder. Redox Biol..

[93] Lo C.H., Zeng J. (2023). Defective lysosomal acidification: A new prognostic marker and therapeutic target for neurodegenerative diseases. Transl. Neurodegener..

[94] Querfurth H., Lee H.K. (2021). Mammalian/mechanistic target of rapamycin (mTOR) complexes in neurodegeneration. Mol. Neurodegener..

[95] Tian X., Teng J., Chen J. (2021). New insights regarding SNARE proteins in autophagosome-lysosome fusion. Autophagy.

[96] Lee J.H., Yang D.S., Goulbourne C.N., Im E., Stavrides P., Pensalfini A., Chan H., Bouchet-Marquis C., Bleiwas C., Berg M.J. (2022). Faulty autolysosome acidification in Alzheimer’s disease mouse models induces autophagic build-up of Aβ in neurons, yielding senile plaques. Nat. Neurosci..

[97] Wu L., Lin Y., Song J., Li L., Rao X., Wan W., Wei G., Hua F., Ying J. (2023). TMEM175: A lysosomal ion channel associated with neurological diseases. Neurobiol. Dis..

[98] Jiang Y., Sato Y., Im E., Berg M., Bordi M., Darji S., Kumar A., Mohan P.S., Bandyopadhyay U., Diaz A. (2019). Lysosomal Dysfunction in Down Syndrome Is APP-Dependent and Mediated by APP-βCTF (C99). J. Neurosci..

[99] Lee J.H., Yu W.H., Kumar A., Lee S., Mohan P.S., Peterhoff C.M., Wolfe D.M., Martinez-Vicente M., Massey A.C., Sovak G. (2010). Lysosomal proteolysis and autophagy require presenilin 1 and are disrupted by Alzheimer-related PS1 mutations. Cell.

[100] Jinn S., Drolet R.E., Cramer P.E., Wong A.H., Toolan D.M., Gretzula C.A., Voleti B., Vassileva G., Disa J., Tadin-Strapps M. (2017). TMEM175 deficiency impairs lysosomal and mitochondrial function and increases α-synuclein aggregation. Proc. Natl. Acad. Sci. USA.

[101] Burbulla L.F., Song P., Mazzulli J.R., Zampese E., Wong Y.C., Jeon S., Santos D.P., Blanz J., Obermaier C.D., Strojny C. (2017). Dopamine oxidation mediates mitochondrial and lysosomal dysfunction in Parkinson’s disease. Science.

[102] Liu X., Wang H., Tian X., Luo Y., Ma M., Zheng Z., Wang Y., Feng S., Wang Q., Xu Z. (2025). Depression exacerbates AD pathology through lactate-dependent activation of microglial Kv1.3 to promote Aβ-containing exosome spreading. J. Neuroinflamm..

[103] Zeng J., Indajang J., Pitt D., Lo C.H. (2025). Lysosomal acidification impairment in astrocyte-mediated neuroinflammation. J. Neuroinflamm..

[104] Lakpa K.L., Khan N., Afghah Z., Chen X., Geiger J.D. (2021). Lysosomal Stress Response (LSR): Physiological Importance and Pathological Relevance. J. Neuroimmune Pharmacol..

[105] Settembre C., Di Malta C., Polito V.A., Garcia Arencibia M., Vetrini F., Erdin S., Erdin S.U., Huynh T., Medina D., Colella P. (2011). TFEB links autophagy to lysosomal biogenesis. Science.

[106] Jeong E., Willett R., Rissone A., La Spina M., Puertollano R. (2024). TMEM55B links autophagy flux, lysosomal repair, and TFE3 activation in response to oxidative stress. Nat. Commun..

[107] Logan T., Simon M.J., Rana A., Cherf G.M., Srivastava A., Davis S.S., Low R.L.Y., Chiu C.L., Fang M., Huang F. (2021). Rescue of a lysosomal storage disorder caused by Grn loss of function with a brain penetrant progranulin biologic. Cell.

[108] Zhang S., Tong M., Zheng D., Huang H., Li L., Ungermann C., Pan Y., Luo H., Lei M., Tang Z. (2023). C9orf72-catalyzed GTP loading of Rab39A enables HOPS-mediated membrane tethering and fusion in mammalian autophagy. Nat. Commun..

[109] Beckers J., Van Damme P. (2024). Toxic gain-of-function mechanisms in C9orf72 ALS-FTD neurons drive autophagy and lysosome dysfunction. Autophagy.

[110] Gallagher M.D., Posavi M., Huang P., Unger T.L., Berlyand Y., Gruenewald A.L., Chesi A., Manduchi E., Wells A.D., Grant S.F.A. (2017). A Dementia-Associated Risk Variant near TMEM106B Alters Chromatin Architecture and Gene Expression. Am. J. Hum. Genet..

[111] Nicot S., Verchère J., Bélondrade M., Mayran C., Bétemps D., Bougard D., Baron T. (2019). Seeded propagation of α-synuclein aggregation in mouse brain using protein misfolding cyclic amplification. FASEB J..

[112] Liu S., Perez P., Sun X., Chen K., Fatirkhorani R., Mammadova J., Wang Z. (2024). MLKL polymerization-induced lysosomal membrane permeabilization promotes necroptosis. Cell Death Differ..

[113] Chou C.C., Vest R., Prado M.A., Wilson-Grady J., Paulo J.A., Shibuya Y., Moran-Losada P., Lee T.T., Luo J., Gygi S.P. (2025). Proteostasis and lysosomal repair deficits in transdifferentiated neurons of Alzheimer’s disease. bioRxiv.

[114] Sjödin S., Brinkmalm G., Öhrfelt A., Parnetti L., Paciotti S., Hansson O., Hardy J., Blennow K., Zetterberg H., Brinkmalm A. (2019). Endo-lysosomal proteins and ubiquitin CSF concentrations in Alzheimer’s and Parkinson’s disease. Alzheimer’s Res. Ther..

[115] Galluzzi L., Vitale I., Aaronson S.A., Abrams J.M., Adam D., Agostinis P., Alnemri E.S., Altucci L., Amelio I., Andrews D.W. (2018). Molecular mechanisms of cell death: Recommendations of the Nomenclature Committee on Cell Death 2018. Cell Death Differ.

[116] Aits S., Jäättelä M. (2013). Lysosomal cell death at a glance. J. Cell Sci..

[117] Dowdle W.E., Nyfeler B., Nagel J., Elling R.A., Liu S., Triantafellow E., Menon S., Wang Z., Honda A., Pardee G. (2014). Selective VPS34 inhibitor blocks autophagy and uncovers a role for NCOA4 in ferritin degradation and iron homeostasis in vivo. Nat. Cell Biol..

[118] Dixon S.J., Stockwell B.R. (2014). The role of iron and reactive oxygen species in cell death. Nat. Chem. Biol..

[119] Platt F.M., d’Azzo A., Davidson B.L., Neufeld E.F., Tifft C.J. (2018). Lysosomal storage diseases. Nat. Rev. Dis. Primers.

[120] Henn D., Yang X., Li M. (2025). Lysosomal quality control Review. Autophagy.

[121] Aflaki E., Westbroek W., Sidransky E. (2017). The Complicated Relationship between Gaucher Disease and Parkinsonism: Insights from a Rare Disease. Neuron.

[122] Shemesh E., Deroma L., Bembi B., Deegan P., Hollak C., Weinreb N.J., Cox T.M. (2015). Enzyme replacement and substrate reduction therapy for Gaucher disease. Cochrane Database Syst. Rev..

[123] Donida B., Raabe M., Tauffner B., de Farias M.A., Machado A.Z., Timm F., Kessler R.G., Hammerschmidt T.G., Reinhardt L.S., Brito V.B. (2020). Nanoparticles containing β-cyclodextrin potentially useful for the treatment of Niemann-Pick C. J. Inherit. Metab. Dis..

[124] Crivaro A.N., Ceci R., Boztepe T., Cisneros J.S., Chain C.Y., Huck-Iriart C., Lamas D.G., Islan G.A., Rozenfeld P. (2025). Effective encapsulation of therapeutic recombinant enzyme into polymeric nanoparticles as a potential vehicle for lysosomal disease treatment. Int. J. Biol. Macromol..

[125] Kwatra M., Kwak G., Li H., Suk J.S., Ko H.S. (2025). Polymeric nanoparticle-mediated GBA1 gene therapy is neuroprotective in a preclinical model of Parkinson’s disease. Drug Deliv. Transl. Res..

[126] Parenti G. (2009). Treating lysosomal storage diseases with pharmacological chaperones: From concept to clinics. EMBO Mol. Med..

[127] Yamashima T. (2023). Implication of Vegetable Oil-Derived Hydroxynonenal in the Lysosomal Cell Death for Lifestyle-Related Diseases. Nutrients.

[128] Houglum K., Filip M., Witztum J.L., Chojkier M. (1990). Malondialdehyde and 4-hydroxynonenal protein adducts in plasma and liver of rats with iron overload. J. Clin. Investig..

[129] Mahapatra K.K., Mishra S.R., Behera B.P., Patil S., Gewirtz D.A., Bhutia S.K. (2021). The lysosome as an imperative regulator of autophagy and cell death. Cell. Mol. Life Sci..

[130] Radulovic M., Yang C., Stenmark H. (2025). Lysosomal membrane homeostasis and its importance in physiology and disease. Nat. Rev. Mol. Cell Biol..

[131] Alexander A., Cai S.L., Kim J., Nanez A., Sahin M., MacLean K.H., Inoki K., Guan K.L., Shen J., Person M.D. (2010). ATM signals to TSC2 in the cytoplasm to regulate mTORC1 in response to ROS. Proc. Natl. Acad. Sci. USA.

[132] Li L., Sun S., Tan L., Wang Y., Wang L., Zhang Z., Zhang L. (2019). Correction to “Polystyrene Nanoparticles Reduced ROS and Inhibited Ferroptosis by Triggering Lysosome Stress and TFEB Nucleus Translocation in a Size-Dependent Manner”. Nano Lett..

[133] Miwa S., Kashyap S., Chini E., von Zglinicki T. (2022). Mitochondrial dysfunction in cell senescence and aging. J. Clin. Investig..

[134] Hanslik K.L., Ulland T.K. (2020). The Role of Microglia and the Nlrp3 Inflammasome in Alzheimer’s Disease. Front. Neurol..

[135] Simpson D.S.A., Oliver P.L. (2020). ROS Generation in Microglia: Understanding Oxidative Stress and Inflammation in Neurodegenerative Disease. Antioxidants.

[136] Halcrow P.W., Lynch M.L., Geiger J.D., Ohm J.E. (2021). Role of endolysosome function in iron metabolism and brain carcinogenesis. Semin. Cancer Biol..

[137] Anandhan A., Dodson M., Shakya A., Chen J., Liu P., Wei Y., Tan H., Wang Q., Jiang Z., Yang K. (2023). NRF2 controls iron homeostasis and ferroptosis through HERC2 and VAMP8. Sci. Adv..

[138] Rizzollo F., More S., Vangheluwe P., Agostinis P. (2021). The lysosome as a master regulator of iron metabolism. Trends Biochem. Sci..

[139] Kurz T., Eaton J.W., Brunk U.T. (2010). Redox activity within the lysosomal compartment: Implications for aging and apoptosis. Antioxid. Redox Signal.

[140] Chen Y., Fang Z.M., Yi X., Wei X., Jiang D.S. (2023). The interaction between ferroptosis and inflammatory signaling pathways. Cell Death Dis..

[141] Chen L., Shen Q., Liu Y., Zhang Y., Sun L., Ma X., Song N., Xie J. (2025). Homeostasis and metabolism of iron and other metal ions in neurodegenerative diseases. Signal Transduct. Target. Ther..

[142] Huang B., Wang H., Liu S., Hao M., Luo D., Zhou Y., Huang Y., Nian Y., Zhang L., Chu B. (2025). Palmitoylation-dependent regulation of GPX4 suppresses ferroptosis. Nat. Commun..

[143] Xue Q., Yan D., Chen X., Li X., Kang R., Klionsky D.J., Kroemer G., Chen X., Tang D., Liu J. (2023). Copper-dependent autophagic degradation of GPX4 drives ferroptosis. Autophagy.

[144] Wang L.Q., Ma Y., Zhang M.Y., Yuan H.Y., Li X.N., Xia W., Zhao K., Huang X., Chen J., Li D. (2024). Amyloid fibril structures and ferroptosis activation induced by ALS-causing SOD1 mutations. Sci. Adv..

[145] Wang Y., Lv M.N., Zhao W.J. (2023). Research on ferroptosis as a therapeutic target for the treatment of neurodegenerative diseases. Ageing Res. Rev..

[146] Lloyd-Evans E., Waller-Evans H. (2020). Lysosomal Ca^2+^ Homeostasis and Signaling in Health and Disease. Cold Spring Harb. Perspect. Biol..

[147] Hui L., Geiger N.H., Bloor-Young D., Churchill G.C., Geiger J.D., Chen X. (2015). Release of calcium from endolysosomes increases calcium influx through N-type calcium channels: Evidence for acidic store-operated calcium entry in neurons. Cell Calcium.

[148] Peng W., Wong Y.C., Krainc D. (2020). Mitochondria-lysosome contacts regulate mitochondrial Ca^2+^ dynamics via lysosomal TRPML1. Proc. Natl. Acad. Sci. USA.

[149] Feng X., Cai W., Li Q., Zhao L., Meng Y., Xu H. (2025). Activation of lysosomal Ca^2+^ channels mitigates mitochondrial damage and oxidative stress. J. Cell Biol..

[150] Cisneros J., Belton T.B., Shum G.C., Molakal C.G., Wong Y.C. (2022). Mitochondria-lysosome contact site dynamics and misregulation in neurodegenerative diseases. Trends Neurosci..

[151] Kiraly S., Stanley J., Eden E.R. (2025). Lysosome-Mitochondrial Crosstalk in Cellular Stress and Disease. Antioxidants.

[152] Yuan Z., Li Y., Sun M., Yuan M., Han Z., Li X., Liu S., Sun Y., Cao J., Li F. (2025). Recent progress in ROS-responsive biomaterials for the diagnosis and treatment of cardiovascular diseases. Theranostics.

[153] Hoang T.T., Smith T.P., Raines R.T. (2017). A Boronic Acid Conjugate of Angiogenin that Shows ROS-Responsive Neuroprotective Activity. Angew. Chem. Int. Ed. Engl..

[154] Chen Y., Yang X., Li J., Luo H., Huang Q., Yang W., Lei T., Lui S., Gong Q., Li H. (2025). A nasally administrated reactive oxygen species-responsive carrier-free gene delivery nanosystem for Alzheimer’s disease combination therapy. J. Control. Release.

[155] Xu H., Liu Y. (2023). ROS-responsive nanomodulators downregulate IFITM3 expression and eliminate ROS for Alzheimer’s disease combination treatment. J. Colloid Interface Sci..

[156] Yang P., Li Y., Qian K., Zhou L., Cheng Y., Wu J., Xu M., Wang T., Yang X., Mu Y. (2024). Precise Modulation of Pericyte Dysfunction by a Multifunctional Nanoprodrug to Ameliorate Alzheimer’s Disease. ACS Nano.

[157] Guo Q., Wang T., Qian C., Wang X. (2024). Redox Oxygen Species-Responsive Nanotheranostics with Dual-Channel Fluorescent Turn-On for Early Diagnosis and Targeted Therapy of Alzheimer’s Disease. Small.

[158] Yang Y., Li Z., Fan X., Jiang C., Wang J., Rastegar-Kashkooli Y., Wang T.J., Wang J., Wang M., Cheng N. (2024). Nanozymes: Potential Therapies for Reactive Oxygen Species Overproduction and Inflammation in Ischemic Stroke and Traumatic Brain Injury. ACS Nano.

[159] Bi X., Cao N., He J. (2024). Recent advances in nanoenzymes for Alzheimer’s disease treatment. Colloids Surf. B Biointerfaces.

[160] Liang M., Yan X. (2019). Nanozymes: From New Concepts, Mechanisms, and Standards to Applications. Acc. Chem. Res..

[161] Feng W., Han X., Hu H., Chang M., Ding L., Xiang H., Chen Y., Li Y. (2021). 2D vanadium carbide MXenzyme to alleviate ROS-mediated inflammatory and neurodegenerative diseases. Nat. Commun..

[162] Li B., Bai Y., Yion C., Wang H., Su X., Feng G., Guo M., Peng W., Shen B., Zheng B. (2023). Single-Atom Nanocatalytic Therapy for Suppression of Neuroinflammation by Inducing Autophagy of Abnormal Mitochondria. ACS Nano.

[163] Kwon H.J., Kim D., Seo K., Kim Y.G., Han S.I., Kang T., Soh M., Hyeon T. (2018). Ceria Nanoparticle Systems for Selective Scavenging of Mitochondrial, Intracellular, and Extracellular Reactive Oxygen Species in Parkinson’s Disease. Angew. Chem. Int. Ed. Engl..

[164] Li L., Lu Y., Xu X., Yang X., Chen L., Jiang C., Wang Y., Hu W., Wei X., Yang Z. (2021). Catalytic-Enhanced Lactoferrin-Functionalized Au-Bi_2_Se_3_ Nanodots for Parkinson’s Disease Therapy via Reactive Oxygen Attenuation and Mitochondrial Protection. Adv. Healthc. Mater..

[165] Jia Z., Yuan X., Wei J.-a., Guo X., Gong Y., Li J., Zhou H., Zhang L., Liu J. (2021). A Functionalized Octahedral Palladium Nanozyme as a Radical Scavenger for Ameliorating Alzheimer’s Disease. ACS Appl. Mater. Interfaces.

[166] Song X., Ding Q., Wei W., Zhang J., Sun R., Yin L., Liu S., Pu Y. (2023). Peptide-Functionalized Prussian Blue Nanomaterial for Antioxidant Stress and NIR Photothermal Therapy against Alzheimer’s Disease. Small.

[167] Tian J., Peng Q., Shen Y., Liu X., Li D., Li J., Guo S., Meng C., Xiao Y. (2024). Chondroitin sulphate modified MoS_2_ nanoenzyme with multifunctional activities for treatment of Alzheimer’s disease. Int. J. Biol. Macromol..

[168] Li L., Xiong Y., Zhang Y., Yan Y., Zhao R., Yang F., Xie M. (2024). Biofilm-camouflaged Prussian blue synergistic mitochondrial mass enhancement for Alzheimer’s disease based on Cu^2+^ chelation and photothermal therapy. J. Control. Release.

[169] Shan Q., Zhi Y., Chen Y., Yao W., Zhou H., Che J., Bai F. (2024). Intranasal liposomes co-delivery of Aβ-targeted KLVFF and ROS-responsive ceria for synergistic therapy of Alzheimer’s disease. Chem. Eng. J..

[170] Ge K., Mu Y., Liu M., Bai Z., Liu Z., Geng D., Gao F. (2022). Gold Nanorods with Spatial Separation of CeO_2_ Deposition for Plasmonic-Enhanced Antioxidant Stress and Photothermal Therapy of Alzheimer’s Disease. ACS Appl. Mater. Interfaces.

[171] Varesi A., Campagnoli L.I.M., Carrara A., Pola I., Floris E., Ricevuti G., Chirumbolo S., Pascale A. (2023). Non-Enzymatic Antioxidants against Alzheimer’s Disease: Prevention, Diagnosis and Therapy. Antioxidants.

[172] He M., Zhang X., Ran X., Zhang Y., Nie X., Xiao B., Lei L., Zhai S., Zhu J., Zhang J. (2024). Black Phosphorus Nanosheets Protect Neurons by Degrading Aggregative α-syn and Clearing ROS in Parkinson’s Disease. Adv. Mater..

[173] Guo W., Ji M., Li Y., Qian M., Qin Y., Li W., Nie H., Lv W., Jiang G., Huang R. (2024). Iron ions-sequestrable and antioxidative carbon dot-based nano-formulation with nitric oxide release for Parkinson’s disease treatment. Biomaterials.

[174] Qi X., Li L., Ye P., Xie M. (2024). Macrophage Membrane-Modified MoS_2_ Quantum Dots as a Nanodrug for Combined Multi-Targeting of Alzheimer’s Disease. Adv. Healthc. Mater..

[175] Kosyakovsky J., Fine J.M., Frey W.H., Hanson L.R. (2021). Mechanisms of Intranasal Deferoxamine in Neurodegenerative and Neurovascular Disease. Pharmaceuticals.

[176] Weinreb O., Mandel S., Youdim M.B.H., Amit T. (2013). Targeting dysregulation of brain iron homeostasis in Parkinson’s disease by iron chelators. Free Radic. Biol. Med..

[177] Hanson L.R., Fine J.M., Renner D.B., Svitak A.L., Burns R.B., Nguyen T.M., Tuttle N.J., Marti D.L., Panter S.S., Frey W.H. (2012). Intranasal delivery of deferoxamine reduces spatial memory loss in APP/PS1 mice. Drug Deliv. Transl. Res..

[178] Febbraro F., Andersen K.J., Sanchez-Guajardo V., Tentillier N., Romero-Ramos M. (2013). Chronic intranasal deferoxamine ameliorates motor defects and pathology in the α-synuclein rAAV Parkinson’s model. Exp. Neurol..

[179] Lei L., Yuan J., Dai Z., Xiang S., Tu Q., Cui X., Zhai S., Chen X., He Z., Fang B. (2024). Targeting the Labile Iron Pool with Engineered DFO Nanosheets to Inhibit Ferroptosis for Parkinson’s Disease Therapy. Adv. Mater..

[180] You L., Wang J., Liu T., Zhang Y., Han X., Wang T., Guo S., Dong T., Xu J., Anderson G.J. (2018). Targeted Brain Delivery of Rabies Virus Glycoprotein 29-Modified Deferoxamine-Loaded Nanoparticles Reverses Functional Deficits in Parkinsonian Mice. ACS Nano.

[181] Zeng J., Acin-Perez R., Assali E.A., Martin A., Brownstein A.J., Petcherski A., Fernández-Del-Rio L., Xiao R., Lo C.H., Shum M. (2023). Restoration of lysosomal acidification rescues autophagy and metabolic dysfunction in non-alcoholic fatty liver disease. Nat. Commun..

[182] Behzadi S., Serpooshan V., Tao W., Hamaly M.A., Alkawareek M.Y., Dreaden E.C., Brown D., Alkilany A.M., Farokhzad O.C., Mahmoudi M. (2017). Cellular uptake of nanoparticles: Journey inside the cell. Chem. Soc. Rev..

[183] Kong W., Wei Y., Dong Z., Liu W., Zhao J., Huang Y., Yang J., Wu W., He H., Qi J. (2024). Role of size, surface charge, and PEGylated lipids of lipid nanoparticles (LNPs) on intramuscular delivery of mRNA. J. Nanobiotechnol..

[184] Albanese A., Tang P.S., Chan W.C. (2012). The effect of nanoparticle size, shape, and surface chemistry on biological systems. Annu. Rev. Biomed. Eng..

[185] Feng Y., Fu H., Zhang X., Liu S., Wei X. (2024). Lysosome toxicities induced by nanoparticle exposure and related mechanisms. Ecotoxicol. Environ. Saf..

[186] Abulikemu A., Zhao X., Qi Y., Liu Y., Wang J., Zhou W., Duan H., Li Y., Sun Z., Guo C. (2022). Lysosomal impairment-mediated autophagy dysfunction responsible for the vascular endothelial apoptosis caused by silica nanoparticle via ROS/PARP1/AIF signaling pathway. Environ. Pollut..

[187] Dostert C., Pétrilli V., Van Bruggen R., Steele C., Mossman B.T., Tschopp J. (2008). Innate immune activation through Nalp3 inflammasome sensing of asbestos and silica. Science.

[188] Lo C.H., O’Connor L.M., Loi G.W.Z., Saipuljumri E.N., Indajang J., Lopes K.M., Shirihai O.S., Grinstaff M.W., Zeng J. (2024). Acidic Nanoparticles Restore Lysosomal Acidification and Rescue Metabolic Dysfunction in Pancreatic β-Cells under Lipotoxic Conditions. ACS Nano.

[189] Arotcarena M.L., Soria F.N., Cunha A., Doudnikoff E., Prévot G., Daniel J., Blanchard-Desce M., Barthélémy P., Bezard E., Crauste-Manciet S. (2022). Acidic nanoparticles protect against α-synuclein-induced neurodegeneration through the restoration of lysosomal function. Aging Cell.

[190] Bourdenx M., Daniel J., Genin E., Soria F.N., Blanchard-Desce M., Bezard E., Dehay B. (2016). Nanoparticles restore lysosomal acidification defects: Implications for Parkinson and other lysosomal-related diseases. Autophagy.

[191] Prévot G., Soria F.N., Thiolat M.L., Daniel J., Verlhac J.B., Blanchard-Desce M., Bezard E., Barthélémy P., Crauste-Manciet S., Dehay B. (2018). Harnessing Lysosomal pH through PLGA Nanoemulsion as a Treatment of Lysosomal-Related Neurodegenerative Diseases. Bioconjugate Chem..

[192] Xu L., Wu X., Zhao S., Hu H., Wang S., Zhang Y., Chen J., Zhang X., Zhao Y., Ma R. (2024). Harnessing Nanochaperone-Mediated Autophagy for Selective Clearance of Pathogenic Tau Protein in Alzheimer’s Disease. Adv. Mater..

[193] Tedeschi V., Nele V., Valsecchi V., Anzilotti S., Vinciguerra A., Zucaro L., Sisalli M.J., Cassiano C., De Iesu N., Pignataro G. (2024). Nanoparticles encapsulating phosphatidylinositol derivatives promote neuroprotection and functional improvement in preclinical models of ALS via a long-lasting activation of TRPML1 lysosomal channel. Pharmacol. Res..

[194] Vest R.T., Chou C.C., Zhang H., Haney M.S., Li L., Laqtom N.N., Chang B., Shuken S., Nguyen A., Yerra L. (2022). Small molecule C381 targets the lysosome to reduce inflammation and ameliorate disease in models of neurodegeneration. Proc. Natl. Acad. Sci. USA.

[195] Chung C.Y., Shin H.R., Berdan C.A., Ford B., Ward C.C., Olzmann J.A., Zoncu R., Nomura D.K. (2019). Covalent targeting of the vacuolar H^+^-ATPase activates autophagy via mTORC1 inhibition. Nat. Chem. Biol..

[196] Zhai L., Gao Y., Yang H., Wang H., Liao B., Cheng Y., Liu C., Che J., Xia K., Zhang L. (2025). A ROS-Responsive nanoparticle for nuclear gene delivery and autophagy restoration in Parkinson’s disease therapy. Biomaterials.

[197] Xu S., Yang P., Qian K., Li Y., Guo Q., Wang P., Meng R., Wu J., Cao J., Cheng Y. (2022). Modulating autophagic flux via ROS-responsive targeted micelles to restore neuronal proteostasis in Alzheimer’s disease. Bioact. Mater..

[198] Yang Z., Shi H., Cai G., Jiang S., Hu Z., Wang Z. (2023). A Reactive Oxygen Species-Responsive Targeted Nanoscavenger to Promote Mitophagy for the Treatment of Alzheimer’s Disease. Small.

[199] Jiang S., Cai G., Yang Z., Shi H., Zeng H., Ye Q., Hu Z., Wang Z. (2024). Biomimetic Nanovesicles as a Dual Gene Delivery System for the Synergistic Gene Therapy of Alzheimer’s Disease. ACS Nano.

